# A Quantitative Environmental Risk Assessment for Microplastics in Sewage Sludge Applied to Land

**DOI:** 10.1021/acs.est.5c08026

**Published:** 2025-10-20

**Authors:** Paul Boisseaux, Marie Laure Delignette-Muller, Tamara Galloway

**Affiliations:** † Centre for Resilience in Environment, Water and Waste (CREWW), 3286 University of Exeter, North Park Road, Exeter, Devon EX4 4TA, United-Kingdom; ‡ Universite Claude Bernard Lyon 1, LBBE, UMR 5558, CNRS, VetAgro Sup, Villeurbanne 69622, France

**Keywords:** microplastics, species-sensitivity distribution, soil, sludge, risk assessment, 2D Monte Carlo simulations

## Abstract

The application of sewage sludge to land delivers high levels of microplastics (MPs), contributing to soil contamination and chronic effects on soil biota. Despite this, the quantitative chronic risk assessment of MPs in sludge-amended soils (SAS) to soil biota has not been thoroughly addressed, and none has been done directly on sludge samples. Here, we combined environmental exposure and species sensitivity distributions, built on published data, with two-dimensional Monte Carlo simulations to characterize the risk from MPs in sludge samples and in SAS, testing worst-case and realistic case scenarios of sludge behavior in soil. Contamination with MPs frequently exceeded the concentration likely to affect more than 5% of the species. Risk characterization showed that the worst-case MP scenario affects 39% of species, while the realistic scenario affects 15–18% of soil species, implying that the current state of sludge application fails to protect 95% of soil biota. The percentage of species affected by MPs approximately doubled in SAS. Our conservative results suggest that regulatory limits on MPs in sludge being applied to land should be urgently considered.

## Graphical abstract

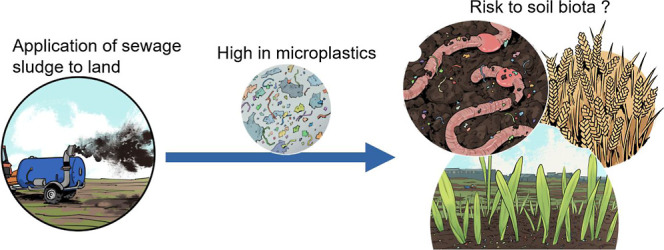

## Introduction

Hundreds of millions of tonnes of sewage sludge are produced globally each year^1^ as a byproduct of wastewater treatment plants (WWTPs) produced during the water depuration process through a series of treatments (e.g., anaerobic digestion or lime stabilization). The stabilized sludge end-product is a solid mud-like substance, or biosolid, rich in organic matter, that is often spread on agricultural lands as a fertilizer, supporting the principles of sustainability^2,3^ and with different restrictions across countries. For example, in the UK, biosolid samples must be analyzed beforehand to ensure they comply with metal concentration thresholds for safe land application. The practice must comply with a biosolid nutrient management matrix, which states what amount of sludge can be applied to land and at what frequency, based on the amount of nitrogen and phosphorus. Depending on the sludge treatment, not all sludge samples can be applied to all types of crops, and some specific patterns of crop rotation must be respected.^4^

A particular feature of sewage sludge is its ability to effectively capture microplastics (MPs) from wastewater. Most WWTPs involving at least a secondary treatment remove the majority (>78%) of MPs from sewage inflow water, typically captured into sludge.^5^ If this sludge is then spread onto land, then the captured MPs will be reintroduced into the environment, where they may persist and accumulate. It has been estimated that between 125 and 850 tonnes of MPs per million inhabitants are returned to agricultural land in Europe alone through the application of sewage sludge each year.^6^ This equates to a yearly application rate of 63 to 430 × 10^3^ tonnes of MPs, a figure that exceeds the total accumulation of 93 to 236 × 10^3^ tonnes of MPs estimated to be floating in the surface waters of the global oceans.^7^

These estimates illustrate the scale of potential applications, but what to do with sludge/biosolids is a complex question. On the one hand, they contain many toxicants or pollutants, such as MPs, organic contaminants, and metals, but on the other hand, they contain nutrients (nitrogen, phosphates, and potassium) that add considerable value to the soil as a fertilizer.^8^ If not recycled, unless new solutions emerge, they are sent to landfills (with potential leakage issues), incinerated, which has additional environmental costs (e.g., atmospheric contamination), composted, or disposed of by other, often inappropriate means.^9^ Finally, recycling to lands provides a financial opportunity for water companies, recognizing this waste product as a valuable resource. For all of these reasons, the recycling of biosolids to land is currently predominant in many developed countries, although there are disparities. For example, around 47–55% of sewage sludge is recycled to land in the USA and 27% across Europe (in certain countries, such as Norway, the figure is approximately 82%; it is 63% in Ireland and 48% in Germany, while in the Netherlands, this is 0%).^1,3,6,9,10^ To our knowledge, there are currently no regulations restricting sludge application based on its MP content.

Soil is of fundamental importance in supporting terrestrial ecosystems and the services they provide, such as decomposition of organic matter and cycling of nutrients and carbon, maintenance of soil structure, gas exchange, and food production.^11^ Ensuring that the application of sludge/biosolids to land, particularly agricultural lands, is safe and preserves the health of soils in the long term is crucial, yet it must take account of the simultaneous presence of both beneficial nutrients and harmful micropollutants. MPs pose a risk to soil ecosystems in many ways. They may impact both abiotic and biotic components of soils. For example, they can alter physical and chemical properties such as soil structure and density, water retention, conductivity, and nutrient cycling.^12^ They may also induce ecotoxicological effects on soil biota and modify enzyme activity and stress species. Mild effects or even adverse effects to terrestrial species that perform crucial ecological functions could compromise soil ecosystems, including impacts to soil-dwelling invertebrates, fungi, bacteria, and plants.^13^ As a result, the health and good functioning of these ecosystems could be impaired. It could lead to ecological surprises.^14^

Performing risk assessment (RA) for MPs is hampered by the variability and complexity of the data and statistical methods employed. Different factors that may be taken into account include qualitative or quantitative data on fate and behavior, abundance, migration, and biological or ecological impacts and interactions in different habitats,^15–17^ as well as the way effect thresholds and environmental concentrations are compared and computed. As examples of this heterogeneity, some experts construct species sensitivity distributions (SSDs) and superimpose environmental exposure distributions (EEDs) for direct risk characterization (e.g., ref 18), whereas others use predicted no effect concentrations (PNECs) extracted from SSDs to construct risk ratio distributions.^19,20^ Several models and computer programs exist for constructing SSDs. Some include only acute effects, others only chronic effects, and others a mix of time exposure. Some studies use assessment factors in SSDs, including for time exposure correction (e.g., ref 21), while others do not. SSDs can be constructed for various MP sizes and polymers.^22^ Other experts employ rescaling methods for polydispersity and standard MP size range.^19^ Lithner et al.^23^ devised a hazard index for 55 different polymers, taking into account both monomer type and additive chemicals, a concept further developed by others^24,25^ to categorize risks of MPs in surface waters. Wang et al.^26^ combined different risk indices to account for polymer type, ecological risk, and pollutant behavior and classified the potential ecological risk from MPs in air, water, and soil as low but highlighted the increased indirect risks associated with other complexed pollutants, especially metals. Regarding sewage sludge, extreme levels of hazard were deduced in sludge regional treatment plants in India using a combination of pollution load and polymer hazard indices,^27^ although risks to soil-dwelling species were not explicitly addressed. Another study performed a quantitative RA on global soil invertebrates and plants, with sludge-amended soils (SAS) being assessed as a separate category, for which the proportion at high risk was greater than other types of soil.^19^

It is extremely important for the environment and human health globally to have a comprehensive framework to address the risks posed to land by the practice of sewage sludge application.^14,28^ Indeed, sewage sludge is, overall, the most contaminated environmental matrix worldwide (e.g., ref 29). No other types of samples are globally as contaminated by MPs as sewage sludge. Indeed, most MPs are removed from WWTP input wastewater and concentrated into sludge during the depuration and stabilization process (e.g., see concentration ranges for anaerobically digested and lime-stabilized sludges, ref 29). Stabilized sewage sludge is extensively applied to land and contaminates soils, notwithstanding that most of them are applied to agricultural lands, therefore potentially impacting crops and food for human consumption. A few scarce studies addressed the RA of MPs in various soil types, including SAS, but only one study addressed it as a separate category of other soils.^19^ Several aspects remain unaddressed, such as the direct consideration of sludge samples, the inclusion of nonsevere end points, the inclusion of microorganisms, the adjustment for long-term effects, and the uncertainty in both exposure and effect data for risk characterization. The breadth of the problem, the complexity of data, the lack of multiple studies/perspectives, and the heterogeneity in statistical methods emphasize the need for urgent research in this area. To date, there is no study that is entirely dedicated to the RA of MPs in SAS. None focused on potential chronic effects on nonsevere end points. None has addressed sludge samples directly.

We address this knowledge gap by combining data from published articles with more data than any previous study on this specific topic. EED of MPs in sludge samples as well as in SAS was built separately. In order to reflect potential effects on the long term, we focused on equivalent chronic effect thresholds on nonsevere end points. From the SSD, the hazard concentration inducing effects on 5% of species (HC_5_) was derived as a safe concentration, ensuring the safety of 95% of species. When the HC_5_ is exceeded, the concentration is considered unsafe. Comparing SSD with the EED of MPs in stabilized sewage sludge and SAS allows any overlap to be determined and the proportion of safety-compliant samples to be quantified.

A novelty of the present study is the comparison of scenarios for sludge behavior in soil after its application. A worst-case scenario is presented, which assumes that wildlife is exposed to 100% of the sludge MP concentration upon application. A more realistic scenario accounts for the environmental dilution of the sludge concentration over time due to vertical and horizontal migration caused by weather, soil type, biota activity, plowing, and other factors.

An additional novelty of the present study is the use of two-dimensional Monte Carlo (MC2D) simulations to robustly characterize the risk from MPs in sludge directly and in SAS. The SSD and EED characterizing the variability of biological and environmental concentrations from observed data are subject to uncertainty. MC2D takes into account both the variability and the uncertainty of both the whole SSD and the whole EED. While MC2D simulations for RA are employed in the food safety sector^30,31^ and in human toxicology,^32–34^ to our knowledge, this method has never been employed in plastics ecotoxicology, and not in soils, where more approximate methods are usually used. It significantly leverages the technical quality for risk characterization, and the present study fills the method gap. It paves the way for its implementation to quantitatively assess risk using the SSD and EED. As MC2D simulations integrate the whole SSD and EED with their uncertainty, they are more robust than methods focusing solely on point estimates of percentiles of those distributions (such as the HC_5_). HC_5_ is often subject to higher uncertainty, as it lies on the extreme left tail of the distribution, where inclusion or exclusion criteria of individual values can have a substantial impact on the results, and the model selection can result in varying threshold values.

The primary objective of this study was to fill the knowledge gap to comprehensively understand and quantify the risks posed by MPs to soil biota by the application of sludge to lands worldwide. This was performed by quantitatively assessing not only the SAS but also the direct sludge samples that are being applied, which has never been done before. Furthermore, we compare various scenarios for practical understanding of the risks involved. State-of-the-art statistical approaches, never used before in soil plastic ecotoxicology, were employed to ensure high clarity and technical quality, with a script provided for reproducibility and a potential guidance tool for future studies.

## Materials and Methods

### Species Sensitivity Distribution (SSD)

#### Rationale

Various published studies on MPs across multiple species were screened to capture data across several levels of biological organization, from molecular through to whole organism effects. Toxicity end points including the highest observed no effect concentration (HONEC), no observed effect concentration (NOEC), and lowest observed effect concentration (LOEC) were extracted. Severe (e.g., death, reproduction, growth) and nonsevere (e.g., enzyme activity, energy levels) end points were included. These values were then standardized into a unified metric referred to here as **NOEC**_**equivalent**_ to ensure consistency in RA. The NOEC_equivalent_ is defined here as the concentration of MPs that does not exert significant effects on a nonsevere end point over the long term (>41 days, see Table S4). These conservative values were compiled and used to construct an SSD to characterize the hazard of MPs in soils.

#### Studies

We retrieved and combined the study shortlists on SSDs for MPs in soil from two recent comprehensive studies: *n* = 28 papers^20^ and *n* = 37 papers.^18^ Each study was individually screened, and data were individually extracted to ensure complete, independent data collection. Additionally, more stringent exclusion criteria were applied to enhance data quality, as detailed in Table S1. In brief, selected studies were those that assessed the chronic (>5 days) toxicity of MPs (size range 1 μm–5000 μm, excluding biopolymers), expressed MP concentrations as weight per dry weight either in percentage or absolute value (e.g., % w/w or g/kg dw) or number per dry weight (e.g., nb/kg dw), and their effects on soil biota, including plants, fungi, bacteria, and animals other than mammals, all of them living in soils. After curation of studies, we retained 91 entries split across 25 “species” and 37 separate publications.

#### Descriptor Extraction

When multiple exposure times were available, the longer duration was prioritized. Time was converted to 7 or 28 days if it was recorded as 1 or 4 weeks, respectively. One month was converted to 30 days. For studies examining multiple species, a toxicological value was extracted for each species. Similarly, if a study investigated different types or sizes of MPs, then a toxicological value was extracted for each type and size. Consequently, some studies have multiple entries in the data set. Soil microbial end points were included only if they were a focus of the research (e.g., in ref 35, which focused on both soil microbiota and *Metaphire guillelmi* microbiota). If soil microbes were a peripheral aspect of the study (e.g., if the research primarily focused on plants, as in ref 36), they were not considered. In most cases, effects were considered without multiple stressors,^37^ e.g., the effects on *Aporrectodea rosea* were considered only in unplanted conditions; in ref 38, the effects on *Triticum aestivum* were included only without the coexistence of worms, but where the design was complex, multiple stressors were considered, as for example in ref 39 where inter/intra plant competitions were crossed with MPs.

HONEC, NOEC, and LOEC were the primary descriptors. Here, we define the LOEC as *sensitivity threshold*. It corresponds to the lowest tested concentration at which a significant difference is observed compared to controls across all assessed end points. This significant difference could be either an increase or a decrease of the end point, compared to controls. The NOEC corresponds to the highest tested concentration at which no significant difference is found compared to controls. The HONEC corresponds to studies where no significant effect at all is detected among any of the tested concentrations compared to controls, and so it corresponds to the highest of these tested concentrations.

Because there was a wide array of studies in different branches of ecotoxicology and ecology on a wide array of species which employ different approaches and tackle complex questions not limited to providing toxicological values, it was impossible to have a strict and homogeneous statistical criteria approach. In the vast majority of studies, the determination of a sensitivity threshold or any other descriptor was based on statistical significance or models. In a few studies, the significant difference to extract the descriptor was determined on the basis of the overall interpretation of the study’s elements rather than solely on statistical p-value significance (see Table S2).

#### Conversion from mg/kg to nb/kg

When the concentration equivalent from weight/weight (w/w) to number/weight was found in the article, it was used; otherwise, it was estimated. If the polymer density was found in the article, it was used; otherwise, the density of the corresponding polymer from Table S3 was used. If the microplastic was not a fiber, it was assumed to be a microsphere for simplification (i.e., particles, beads, and fragments were considered spherical). For conversion purposes, only two types were considered: fibers and microspheres. The conversion formula in ref 40 was used, with the only difference being that mg/L was entered as mg/kg. In two exceptions, MPs described as membranous or films were classified as film-shaped, and the estimation was conducted based on this shape.

Many articles mentioned that MPs were sieved, for example, through 500 μm meshes. In the laboratory, the sieved fraction often has a distribution, mode, and median lower than the sieve mesh size; although this varies for each type of MP, polymer, and preparation method, the sieved mesh often corresponds to the end of a distribution. For a particle size approximation, we assumed a normal distribution when MPs were sieved through a specific mesh sieve and the median was retained (e.g., 250 μm was retained if particles were sieved through 500 μm and 125 μm if sieved between 200 and 50 μm). Similarly, if only a size range was provided, half was retained (e.g., 125 μm for particles classified as 100–150 μm). If the particle distribution was provided, then the median diameter (d50) was used. If various size class proportions were given, then the estimated MP size was normalized by these proportions. For fibers, lengths and diameters were usually provided. In a few instances, the fiber diameter was not provided, and so it was assumed and retrieved from commonly found sizes.

#### Toxicological Values: Calculation of NOEC_equivalents_

##### Overall Calculation

Data harmonization was performed to enhance the comparability of each descriptor and its “equivalence” to chronic NOECs within the same category of end point. Uncertainty factors (UFs) were applied as in ref 21 but with modifications. Specifically, we used three UFs with an arbitrary geometric progression with a common ratio of 2: (i) time exposure (three levels: 1, 2, and 4), (ii) descriptor precision and extent of effect (three levels: 1, 2, and 4), and (iii) end point category (three levels: 1, 2, and 4). See details in Table S4. Thus, the selected descriptor was divided by these uncertainty factors as NOEC_equivalent_ = descriptor/(UF_T_ × UF_D_ × UF_C_), where UF_T_ is the UF for time exposure, UF_D_ is the UF for the descriptor precision and extent of effect, and UF_C_ is the UF for the end point category.

##### Descriptor Precision and Extent of Effect

If within the study, only the HONEC could be extracted (right-censored value), it was categorized as “NOEC_b”. This usually occurs when only one or very few concentrations are tested and no effect at all is observed. If both NOEC and LOEC could be derived but their concentration interval was ≥10, then the NOEC was also categorized as “NOEC_b”. If their interval was <10, it was categorized as “NOEC_a”. This typically occurs in comprehensive dose–response designs with narrow concentration intervals, which are optimal for deriving toxicological values with a high degree of confidence. See Figure S1 to help the final selection of the descriptor and associated UFs.

If a LOEC was available, but no NOEC could be obtained from the study, then the LOEC was categorized as “LOEC_b” (it is censored somewhere on the left of the NOEC). If both LOEC and NOEC were available, the LOEC was categorized as LOEC_b if the concentration interval was ≥10, and it was categorized as LOEC_a when the concentration interval was <10. In both cases, if available, the NOEC was used (see the diagram in Figure S1).

##### End Point Category

When both NOEC and LOEC were available for a study, species, polymer, or size, the NOEC was selected as the preferred toxicological descriptor. If the NOEC was the same for different end points, such as survival (category C) and enzymatic activity (category A), then the NOEC from the least severe category was selected, i.e., the one from category A (enzymatic activity) was retained (see diagram in Figure S1). This approach ensures that overly conservative uncertainty factors are not applied. This assumes that end points from different categories have severities in the order A < B < C, with survival being the most severe end point at an individual scale. It is more informative to know that a concentration has no effect on a less severe end point when this absence of effect also applies to a more severe end point at the same concentration. The most severe end points would generally be affected after less severe end points.

End point category C was designated for survival, while category B included end points like reproduction, morphology, growth, biomass, and germination. Note that even for plants, survival is sometimes assessed (e.g., ref 39). We found it more coherent to include germination in category B, aligning it with reproduction-related end points, similar to how hatching success would be categorized for animals. End point category A is the least severe category. It included measures such as enzyme activity, molecular responses, feces excretion rate, bacterial/fungal diversity, oxidative stress, chlorophyll levels, fungi-mediated water aggregation capacity, and energy reserves.

On the contrary, if the LOEC (as the final descriptor before applying UFs) was the same for different end points, e.g., *survival* and *enzymatic activity*, then the LOEC from the most severe end point was retained, which would be *survival* (category C) in this example (see the diagram in Figure S1). This prevents the use of an underconservative uncertainty factor. In other words, there is no relevance to consider LOEC for enzymatic end points if the same concentration triggers mortality, which must be considered first to characterize the extent of hazard.

### Environmental Exposure Distributions (EEDs)

#### Data Selection

Studies of microplastic concentrations in sewage sludge were retrieved from ref 29, which summarized all sludge MP concentrations published to date (last search performed March 2022 as stated by the article). Only entries in nb/weight (dry) were included; entries in other units were excluded. Each study was screened individually to ensure independent data collection and coherence for the purpose of the present study. If necessary, WebPlotDigitizer was used to extract values from the graph. Entries for a type of sludge that was not stabilized or for which no clue in the description was found about whether it could be applied to land were excluded. For example, terms that were excluded included “activated sludge”, “dewatered sludge”, “drained sludge”, and “primary and secondary sludge”. Apart from a few exceptions, a nonexhaustive list of sludge types which were included is “digested sludge”, “thermally dried sludge”, “anaerobically digested sludge”, “lime-stabilized sludge”, “stabilized and dewatered sludge”, “thermophilic composted sludge”, “biosolids”, “secondary digested sludge”, “composting sludge”, “tertiary sludge cake”, and “final dehydrated sludge”. In a study, when several sludge samples were quantified at the same plant, one entry was retained per WWTP as the average of samples at this plant (regardless of normality). In one instance, 28 WWTPs were quantified.^41^ To avoid giving too much weight and creating an imbalance of entries in the data set from this study, it was decided to extract nine recalculated entries corresponding to the percentiles from 10th to 90th by increments of 10th percentiles (median bootstrap estimates from a log–normal fit) to represent the findings but with 9 entries included in the data set, instead of 28. In total, we retained 74 entries of MP data in stabilized sludge that are potentially spread to land around the world, split across 25 articles.

For the SAS, studies were searched in Google Scholar and Web of Knowledge with keywords such as “biosolids”, “sludge”, “spread”, “soil”, and “microplastics”. Each study was screened and then included only if there was clear identification that one of the land areas was treated with sewage sludge. When this was not sufficiently explained, the study was excluded because MP contamination can occur from an array of sources other than sludge. Control fields were included only when within the same study at least one SAS was assessed in parallel. This increased confidence that the authors had selected the control sites specifically for comparison to fields that had received sludge. Two exceptions were made for two studies that, while not assessing SAS, clearly stated the sites had never been treated with sludge, and so they were included as control fields (see Table S5). When several concentrations were provided for the same field, they were averaged so that each entry corresponded to one land area, except if controls were provided for a specific soil layer, in which case the same layer was retained for the SAS comparison and not averaged. If the soil was measured at, for example, 0–5 cm, 5–10 cm, and 0–10 cm, only the overall layer of 0–10 cm was used (e.g., in ref 42). Generally speaking, we tried to have coherence in the depth of soil samples MP concentration extracted, but sometimes it was not possible, as in ref 43, where one field had surface and deep cores, another field had surface only, and the control field had deep cores only. If several fields were sampled but each individual field’s data was not presented (e.g., boxplot without visible individual values), then only the described value(s) in the text were extracted (e.g., median or mean). When considered necessary (e.g., no values at all provided in the text to describe the graph), WebPlotDigitizer was used to extract values from the graph. One study was excluded because the last sludge application was 34 years ago.^44^ In total, 73 entries were retained, 50 SAS and 23 control fields, split across 19 articles (Table S5).

#### Scenarios

The **worst-case scenario** assumed exposure to 100% of the initial sludge MP concentration. However, sludge disperses over time due to various factors. The **realistic scenario** considered a dilution factor of the initial sludge application to assess the long-term safety. To estimate this, the 50th percentile of the EED of sludge was divided by the 50th percentile of the EED of SAS, resulting in a factor of 17, i.e., approximately 6% of its initial concentration. This suggests that the MP concentration in sludge is diluted roughly 17 times over time. Consequently, the EED diluted sludge was calculated as EED sludge divided by 17 for a more realistic RA.

### Data Analysis and Graphs

All data and graphs were processed with the *R* software.^45^ For SSD, a geometric mean was applied by species as per the formula geomean = exp­(mean­(log­(*X*))), where *X* is the descriptor (i.e., NOEC_equivalent_), mean is the average applied to the same species across multiple studies, log is the natural logarithm, and exp is the exponential. Note that for simplification and to cope with the fact that a small number of studies may include plentiful bacterial and fungal “species”, it was decided to treat all “bacteria” as being one species, as for all “fungi” as another “species”. It may underestimate the differences in biodiversity and impact the SSD but prevents the skew that could be caused by including too many species from a limited number of studies. When bacteria or fungi were assessed in an animal (e.g., in the gut of worms), they were considered as an end point instead of a target species (e.g., worm gut dysbiosis where the species is “worm”). When bacteria or fungi were analyzed directly in soil, they were considered as a target species where the species was either bacteria or fungi.

SSD and EED models were built with the *actuar* ^46^ and *fitdistrplus* ^47^ packages. For SSD, the log–normal, log–logistic, and Burr distribution models were assessed on NOEC_equivalent_ data after calculation of the geometric mean per species. These models for SSD are very common (e.g., refs 48 and 49 ). Model selection was based on the lowest AIC (Akaike information criterion). For EED, these three models were tested, and we extended them with Weibull and gamma distributions. Model selection was based on the lowest AIC. Percentiles of SSD and EED were estimated from each selected fitted model, and their 95% confidence intervals were calculated by parametric bootstrap.

For SSD, particular attention was paid to the fifth percentile as it corresponds to the Hazard Concentration for 5% of Species (HC_5_). To be optimistic and considering the feasibility of environmental management, the upper limit of the HC_5_ was calculated here, above which the environmental concentration would be considered unsafe, as they would induce effects on more than 5% of the species.

Risk characterization using MC2D simulations was run using the *mc2d* package.^31,50^ Variability was simulated conditionally to uncertainty for both the effect values (i.e., NOEC_equivalent_) and the MP concentrations in sludge to calculate the proportion of affected species and its uncertainty for different scenarios corresponding to the degree of sludge dilution, from nondilution (worst-case scenario) to a dilution of 17 (realistic scenario). More precisely, for each scenario, the status (affected or not) of 1001 species sampled in the SSD fitted distribution is simulated, taking into account the fitted EED (1001 iterations in the variability dimension). For each of those simulations, the uncertainty on both distributions is taken into account from bootstrap samples (1001 iterations in the uncertainty dimension). From these 1001 × 1001 simulations, the proportion of affected species is calculated by integrating the variability dimension (i.e., taking the mean of the variable coding for the status of each species, 1 if affected, 0 if not). The 1001 simulations of this proportion are used to calculate the uncertainty in this proportion, using its empirical percentiles. MC2D simulations were also run with SAS and control fields data. Data and scripts to fit models and run the MC2D simulations are provided in Supporting Information (file name “*SSD_and_EED_fit*”, available in .pdf and .Rmd).

## Results and Discussion

### EEDs: MPs in Sludge, Sludge-Amended Soils, and Controls

A higher concentration of MPs was observed in SAS than in control fields that did not receive sludge (Figure 1A, *p* < 0.01, Wilcoxon test; Figure 1B, *p* < 0.01, *t*-test after logarithm transformation). Weibull and log–normal distributions were selected for MP concentration in control soils and SAS, respectively (Figure 3). The 50th percentile MP concentration estimate in control fields was 225 MPs/kg [86; 544], while in SAS, it was 1106 MPs/kg [719; 1687] (see Table 1). Raw empirical medians were 159 and 1017 MPs/kg for controls and SAS, respectively. By summarizing all published data to date (*n* = 23 controls, 50 SAS), we demonstrate robustly that SAS have approximately 5–6 times more MPs than control fields. This supports the many studies that report sludge application to significantly increase MP concentrations in soil or acknowledge it is a major MP contributor (e.g., see reviews in refs 51,52 ). Other studies are more nuanced; neither ref 53,54 detected statistically significant differences between biosolids-amended fields (*n* = 4 in ref 53) and control soils. The dynamics, fate, and migration of MPs into the wider environment remain poorly understood. As discussed in ref 55, untreated fields near SAS have been found with high quantities of MPs, suggesting possible direct migration from SAS to their surroundings. The fate of these MPs in soil remains an imperative question to address.

**Figure 1:**
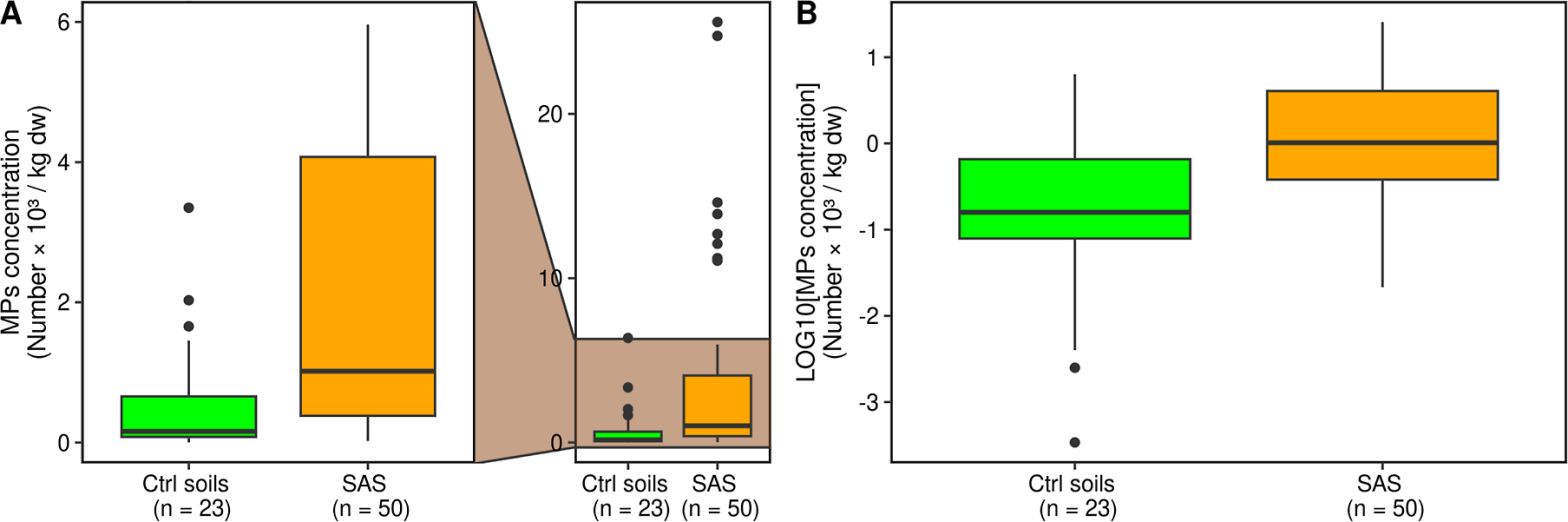
Microplastic concentration in control soils (green) and sludge-amended soils (SAS, orange) expressed as number × 10^3^ per kg of dry weight [A] (*p* < 0.01, Wilcoxon test) and log10-transformed concentrations [B] (*p* < 0.01, two-sample *t*-test).

**Table 1:** Concentration of Microplastics from Relevant Distributions (nb/kg dw)

	percentile	median of bootstrap estimates	two-sided 95% intervals
EED control fields [Weibull model]	5th	2	0; 17
	50th	225	86; 544
	95th	3139	1281; 7425
EED SAS fields [log–normal model]	5th	74	38; 147
	50th	1106	719; 1687
	95th	16,633	8369; 32,672
EED sludge [Burr model]	5th	2865	1786; 4277
	50th	18,923	13,320; 28,519
	95th	1,235,519	324,372; 5,573,397
EED diluted sludge (17-fold dilution) [Burr model]	5th	165	105; 255
	50th	1111	782; 1672
	95th	67,549	21,178; 320,663
SSD [log–normal model]	**5**th (=**HC**_**5**_ **)**	**145**	**13; 1416**
	50th	85,484	19,458; 389,018
	95th	47,202,980	4,336,356; 509,802,430

A Burr distribution was selected for MP concentration in stabilized sludge (Figure 2) and diluted stabilized sludge (Figure 3, assuming a dilution factor of 17). The 50th percentile in EED_sludge_ was 18,923 MPs/kg, while in EED_SAS_, it was 1106 MPs/kg (Table 1). This gave a sludge-to-SAS ratio of 17:1. Thus, as a rough estimate, the MP concentration in soils from land-applied sludge is about 17 times lower than that in the original sludge, i.e., about 6% of the initial 100% concentration. This reflects a realistic environmental scenario compared to the worst-case scenario of long-term exposure to 100% sludge. For comparison, ref 55 observed a dilution ratio of nearly 1:7 when considering the sludge-soil surface/raw sludge (about 14% of initial concentration).

**Figure 2:**
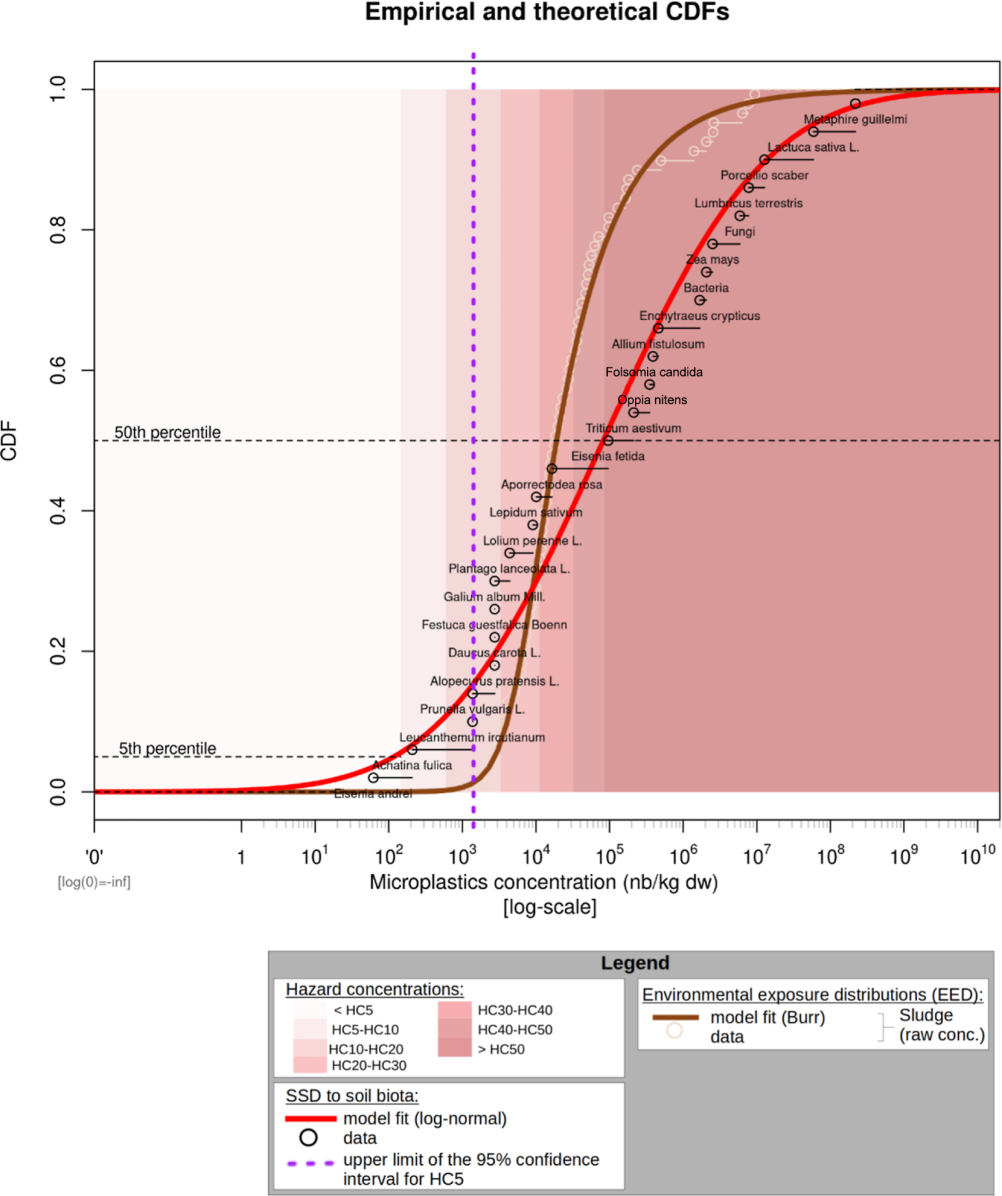
Worst-case scenario of microplastic risk characterization from sludge applied to land. This figure shows the cumulative distribution functions (CDFs) for soil species sensitivity to MPs (SSD, red line) and MP concentrations in undiluted sludge (brown line). The background highlights the range of hazard concentrations, as indicated in the legend, derived from SSD model point estimates. The purple dashed line represents the upper limit of the 95% confidence interval for the HC_5%_ calculated from bootstrap.

**Figure 3:**
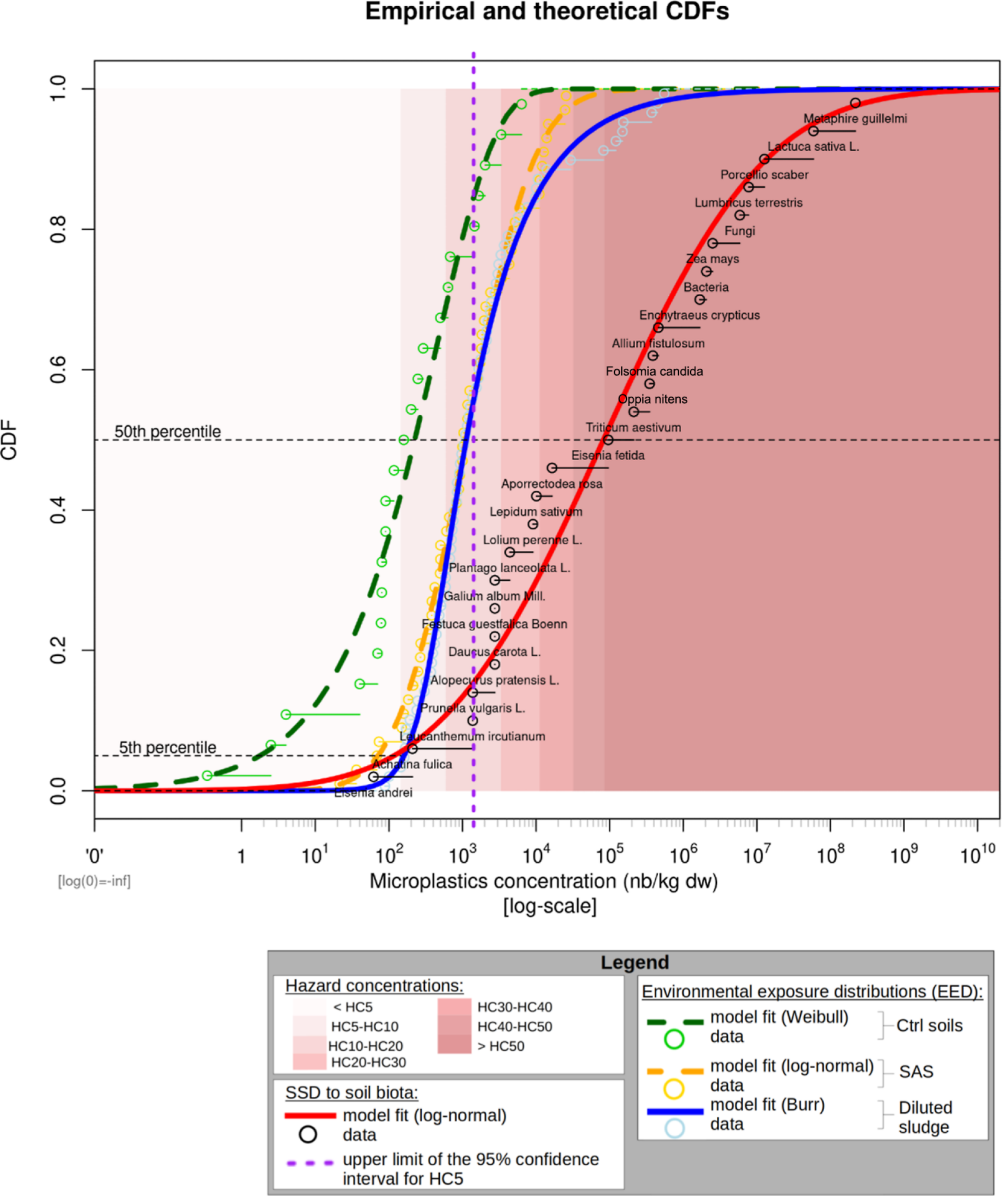
Realistic scenario of microplastic risk characterization of sludge applied to land. This figure shows the cumulative distribution functions (CDFs) for (1) soil species sensitivity to MPs (SSD, red line), (2) MP concentrations in diluted sludge (blue line, 17-fold realistic dilution), (3) MP concentrations in SAS (dashed orange line), and (4) MP concentrations in control soils (dashed green line). The background highlights the range of hazard concentrations, as indicated in the legend, derived from SSD model point estimates. The purple dashed line represents the upper limit for the 95% confidence interval of the HC_5%_ calculated from bootstrap.

In this paper, we focused on quantifying MPs using number per mass (nb/mass) due to its relevance in toxicology and the limited data in other units for environmental samples. However, the variability in methodologies for nb/mass contributes to wide confidence intervals. Indeed, the studies used diverse methods for analyzing MPs (density separation method, nature and size of filter, amount of sample, MP size cutoff lower limit, instrumentation, etc.), which contribute to broad technical variability in the reported concentrations for both toxicity experiments and field studies. This is a recurring feature of MP research. A recommendation for future studies is to (1) when possible, provide both w/w and nb/mass concentrations with detailed methods (e.g., dry or wet weight, any relevant MP behavior such as adherence to containers), (2) characterize MP shape and size distribution, including at least the median, and (3) use additional analytical methods as they become available. For example, recent advancements in thermogravimetric analysis (TGA) and pyrolysis-gas chromatography–mass spectrometry (pyr-GCMS) are expected to generate data, especially for application to environmental samples, with potentially less technical variability than most counting methods used to date. As a result of these recommendations and additional data acquisition, these will allow more reliable comparisons between EED and SSD in the future.

### SSD of MPs on Soil Biota

The distribution selected for the SSD is the log–normal model (Figures 2 and 3). The associated HC_5_ was 145 MPs/kg [13; 1416] (Table 1). This was close to the range reported in ref 18, which found values between 162 and 229 MPs/kg, but several orders of magnitude lower than the HC_5_ of 82,000 MPs/kg suggested in ref 20 and 4 × 10^7^ to 2.3 × 10^8^ MPs/kg dw suggested in ref 19. Differences include the selected descriptor (here we focused on NOEC_equivalent_ as a chronic, nonsevere end point category), the species phylum inclusion or exclusion criteria (we included all types of soil invertebrates as well as plants, bacteria, and fungi), the use of uncertainty factors (we used three types of UFs), the nature of the end points considered, and the inclusion/exclusion of HONECs. Other explaining factors could also be the shortlist of selected studies and the data harmonization method, e.g., use of a correction factor to account for MP polydispersity or MP bioaccessibility.

Model and data selections can also explain differences between studies. For instance, we excluded acute studies and built the SSD based on chronic (≥42 days) NOEC_equivalent_ data for the least severe end points (the most severe end point being death, and the least severe end point category being, e.g., enzymatic activity). Given the diversity of studies, this NOEC_equivalent_ harmonization helps to reduce bias, even though it is not perfect. For example, for bacteria and fungi “species”, their assessed end points could only be in our defined category “A”. When available, measured concentrations were used rather than nominal ones. For MP size, if a study reported particles smaller than 150 μm, we used 75 μm (half of 150 μm) to represent the size, assuming a normal distribution as explained in the Materials and Methods section. Overall, our study offers an additional independent estimate of soil biota sensitivity to MPs, addressing the variability found across the different studies used to build the SSD.

Noteworthy, the HC_5_ of 145 MP/kg [13–1416] dry weight (dw) has a wide confidence interval, with the upper limit of its 95% confidence interval being 1416 MPs/kg. Using the most optimistic approach as well as making environmental management more feasible in the specific context of the present study, considering this upper limit of the confidence interval as the maximum acceptable threshold (1416 MPs/kg or rounded as 1.4 × 10^3^ MPs/kg dw) could be argued. Exceeding this value in the final matrices would indicate that we can probabilistically assume that the practice of sludge application to land is unsafe because it will induce effects on 5% or more species with 95% certainty. Under a worst-case scenario where sludge would remain at its 100% concentration chronically after being spread, the maximum tolerated MP concentration in sludge samples, assuming the receiving soil has 0 MPs/kg, would be 1.4 × 10^3^ MPs/kg dw (upper limit of the HC_5_). When considering a more realistic scenario where soil has a background contamination and a 1:17 dilution would occur after sludge application, 17 times more concentrated MPs could be tolerated in the original sludge sample (i.e., 17 × 1.4 × 10^3^ MPs/kg of dw).

Note, we did not separate the models for fiber and nonfiber MPs or for different size classes of MP. While distinguishing between these types could be highly relevant depending on the context, we opted for a more general approach, considering MPs as a whole. Microfibers are the most abundant MP types reported in sewage across many studies; hence, future analyses may consider these as a separate entity, subject to the availability of a larger number of studies. If future legislation introduces MP limits for sludge application, then our analytical approach could be refined further to include separate limits for different types of MPs.

These SSDs are inherently imperfect due to several factors, such as heterogeneity among study designs, hindering toxicity data extraction, heterogeneity among statistical methods and NOEC criteria for data extraction, interlaboratory variability, heterogeneity of assessed end points and species, extrapolating from laboratory-observed effects to field effects, heterogeneity of MP size distribution, shape, polymers, etc. However, the strength of SSDs lies in their ability to scatter data across various species (25 species in this case) and studies (*n* = 37 here, with *n* = 91 entries). The method used in this paper accounts for the variability in the study designs by applying uncertainty factors.

The studies included in the analysis may refer to species being affected in the long term by a variety of biological end points considered as “nonsevere” (NOEC_equivalent_). “Being affected” could mean experiencing mild effects, and the modulation of each included end point does not necessarily equate to significant toxicity or long-term deleterious effects. Descriptors were extracted regardless of whether effects were increased or decreased compared to controls. These studies also do not account for potential adaptation to pollution. For example, in bacterial diversity, a complete structural change in the community structure can often reflect dysbiosis and ecological harm, whereas significant effects on a single-phylum bacterial change are harder to interpret as toxicity, because potential long-term adaptation may or may not occur. In total, there were 41 entries for end point category A (e.g., enzyme activities, oxidative stress, metal exposure, energy levels, microbial diversity, chlorophyll content, and feces excretion rate), 46 entries for category B (e.g., reproduction and growth), and 4 entries for category C (survival).

For more accurate SSDs, more homogeneous studies are needed with appropriate dose–response designs that allow for precise extraction of toxicological values. Studies must assess effects on a more diverse range of species to address current imbalances. Also, future studies must address the toxicity of MPs specifically in the context of sludge exposure. All studies here focus on MPs in soil alone, but potential synergistic or antagonistic effects could occur with other components in sludge and not just MPs (e.g., metals, pharmaceuticals, organic contaminants, etc.). This will help refine the SSDs.

### Risk Characterization Using Curve Overlaps

Figure 2 shows the cumulative distribution function (CDF) of chronic MPs effect thresholds to soil biota species (SSD). It also displays the EED of MPs in stabilized sludge in a worst-case scenario where it is considered that sludge will not be environmentally diluted over time, and so species will potentially be exposed for long periods of time to 100% of the initial concentration at the surface. The EED_sludge_ and SSD curves significantly overlap, indicating that environmental concentrations correspond to concentrations that affect a high proportion of species. To protect 95% of species, Figure 2 shows that in the most optimistic approach looking at the HC_5_ upper limit (purple line), the sludge distribution has only just started, so practically none of the sludge samples would comply with this safety threshold.

Figure 3 corresponds to a more realistic scenario. It displays the EED of MPs in SAS, in control soils having never received sludge, and in realistically and environmentally diluted sludge, considering a dilution factor closely matching the MP concentrations found in SAS. Here again, the EEDs significantly overlap with the SSD. Therefore, many environmental concentrations correspond to MP concentrations known to affect a high proportion of species, which is concerning. Figure 3 shows that nearly half of the diluted samples (realistic scenario) and half of the SAS would not comply with the safety threshold (i.e., HC_5_ upper limit). Still in Figure 3, we can observe that the safety threshold matches approximately the 80th and 50th percentiles of control and SAS soils, respectively. It means that applying sludge to land would increase by nearly 1/3 the soils failing to meet the safety threshold.

### Risk Characterization Using Simulations

The MC2D simulations provide a robust risk characterization of the practice of sludge application to lands. Their robustness lies in the fact that they integrate not only the variability described in whole distributions of both SSD and EED_sludge_ but also the uncertainty of both. Individual graphs of SSD and EED variabilities with their corresponding uncertainties are provided in Supporting Information. Simulations were repeated under an increasing dilution factor scenario (e.g., 1 for the worst-case and 17 for the realistic scenario). The article script is provided in Supporting Information (file name *SSD_and_EED_fit_article*).

Results of MC2D reveal that MPs in sludge being applied to land may affect 39% [24%; 54%] of soil species under a worst-case scenario (Figure 4 and Table 2). This scenario considers sludge to remain undiluted for long periods of time after land application. However, a realistic scenario considering a dilution of sludge (and its MP content) over time due to natural environmental dilution is more likely. For example, considering a realistic dilution factor of 17 may result in a proportion of affected species of 18% [8%; 30%]. The proportion of affected species for SAS estimated by the simulations is 15% [6%; 27%] (Figure 4 and Table 2).

**Figure 4:**
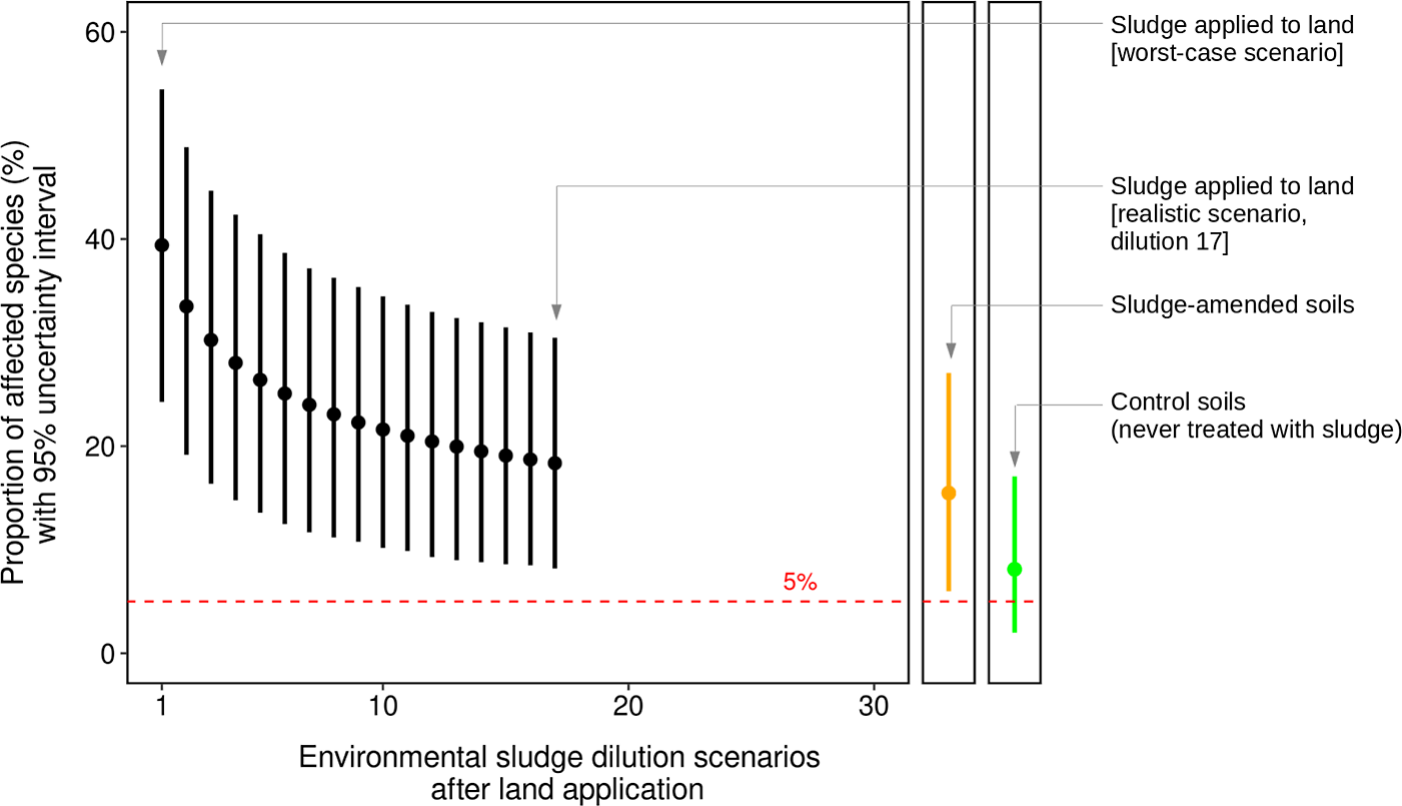
Two-dimensional Monte Carlo simulations (MC2D) on microplastics’ SSD_soil_ and sludge’s microplastics EED_soil_ variability conditionally to uncertainty across various sludge dilution scenarios.

**Table 2:** Estimated Proportion of Affected Species from MC2D Simulations

	point estimate (%)	uncertainty interval [2.5%; 97.5%]
worst-case scenario (undiluted sludge)	39.4	24.3; 54.5
realistic scenario (dilution factor 17)	18.4	08.2; 30.5
SAS	15.5	06.0; 27.1
controls	08.1	02.0; 17.1

Figure 4 shows clearly that in every scenario, from the worst-case to the more realistic ones and SAS, the proportion of affected species is above 5%, even considering 95% uncertainty intervals. Therefore, the MPs contained in sludge that are applied to lands pose an unacceptable risk to soil biota (Figure 5). Interestingly, the proportion of affected species in control soils is 8% [2%; 17%] (Figure 4). Here, the uncertainty intervals of the controls intersect with the 5% threshold. Therefore, some control soils could also affect more than 5% of the species. Overall, under a realistic scenario, the practice of sludge application to land is estimated to double the proportion of affected species by the presence of MPs compared to control soils.

**Figure 5:**
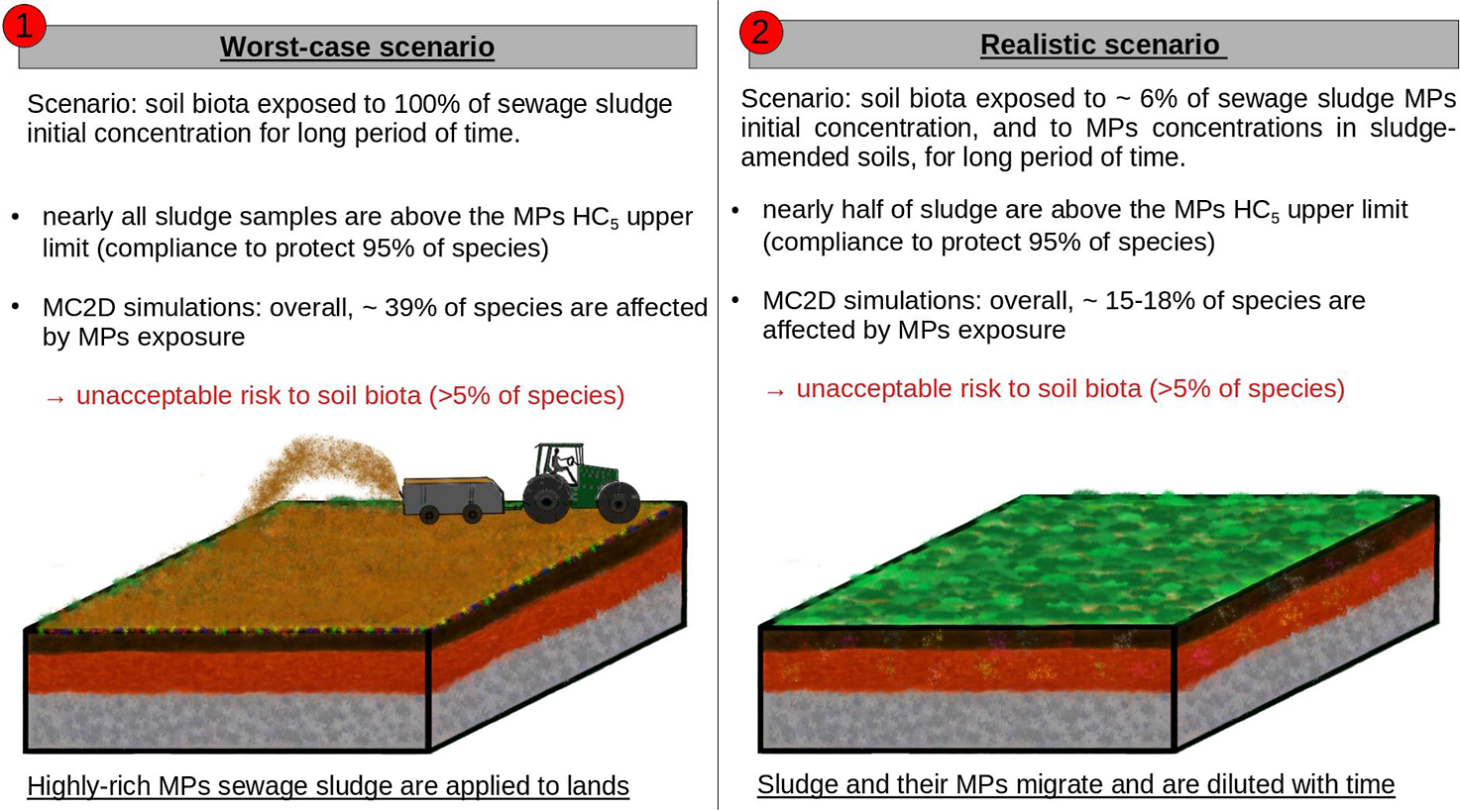
Summary of ecological risks of sludge application to land.

Overall, this paper presents an RA for understanding and quantifying the risks posed to soil biota by the application of sludge containing MPs to land, providing a means for establishing regulatory limits for MPs in sludge used for land application as part of environmental management. Our study confirms that MP concentrations in SAS are significantly higher than those in control fields. Depending on the treatment scenario, applying sludge to soils could add approximately 1/3 of fields to those that exceed the optimistic upper limit thresholds designed to protect 95% of species. Under current global conditions, the sludge-soil continuum is estimated to affect 39% of soil species in a worst-case scenario and 15–18% of species in a realistic scenario in the long term. MPs in control soils are estimated to affect ∼8% of the soil species. The practice of sludge application to land approximately doubles the proportion of affected species resulting from MP exposure. Considering the optimistic upper limit of the HC_5_, safety compliance of sludge samples ranges from none in a worst-case scenario to half of samples in a realistic scenario. Hence, our results infer that methods other than land application would be preferable fates for the majority of sludge samples.

Overall, this paper proposes a preliminary quantitative RA to help sludge management stakeholders and provide guidance to ensure environmental protection.

This figure represents the proportion of affected species (with 95% uncertainty intervals) estimated by MC2D under different dilution scenarios. A dilution factor of 1 represents undiluted sludge (the worst-case scenario), while a dilution factor of 17 corresponds to a more realistic scenario. On the right, the MC2D estimates for SAS (orange) and control soils (green) are displayed.

## Limitations, Suggestions, and Novelty

This study offers valuable insights by comparing scenarios and analyzing sludge samples directly. We recommend similar approaches in future studies for risk characterization, integrating an advanced understanding of sludge fate, which remains insufficiently explored. Improved understanding of MP fate postsludge application, considering soil type, slope, weather, and time, will strengthen exposure assessments. Key knowledge gaps remain, such as migration rates through soil layers, accumulation hotspots, and dose-dependent^56,57^ vs nondose-dependent behaviors.^42,58^ More field studies are needed to refine fate modeling over time and space for better EED construction and creation of dilution scenarios.

The diversity of soil types, properties, and sewage sludge treatments creates potential for complex interactions and coeffects with MPs (e.g., potentially synergistic, antagonistic, linear, nonlinear, monotonic, nonmonotonic). To improve the accuracy of the RA, key soil properties should be identified and prioritized. Future work should further investigate how MPs exert their effects along gradients of soil properties (e.g., organic matter, texture, microbial biomass) and sludge treatments (e.g., lime-stabilized vs anaerobically digested). Studies specifically addressing MP effects in the context of sewage sludge exposure remain scarce. Overall, we still lack a sufficient understanding of how these interacting factors behave. Future studies must progressively fill this knowledge gap to better identify which soil and sludge characteristics are most relevant to include in RA. However, excessive complexity could burden stakeholders and may ultimately be counterproductive, so the challenge is to identify the key cofactors. For example, even under a prioritized scenario where only a few key cofactors are considered, this could lead to a matrix of potentially 144 separate RAs (3 types of sludge stabilization, within which 3 types of soils are considered, within which two types of weather, two types of slope, two MP shapes, and two classes of MP size). The challenge for RA is always to balance the complexity of a real-world situation with the need for pragmatism to generate useful predictions.

In the present quantitative RA, we focused on MPs alone for the SSD and MPs in sewage sludge or SAS for the EED. A key next step is to compile studies assessing the effects of sewage sludge and its embedded MPs under more realistic exposure scenarios, i.e., multiexposure contexts involving MP mixtures, coexposure to chemical pollutants, or direct exposure to sludge containing high contents of organic matter and microbes. This would improve the understanding of interactions among micropollutants and help rank their overall risks within an integrated framework. Future studies should also clarify whether MP-contaminated samples often co-occur with other pollutants of concern (e.g., metals and organic chemicals), in which case targeting highly MP-contaminated samples could simultaneously address multiple pollutant categories.

While fossil-fuel-based MPs are recognized as harmful, our effect thresholds are conservative, focusing on nonsevere end points and chronic exposures, and they account for UFs. Better data will allow refinements, though aligning end points across taxa remains difficult. Toxicity studies should use multiple concentrations (appropriate dose–response design), clear effect gradients, and statistical modeling. A research gap for the future is that SSDs need broader species testing and reference tables reflecting realistic soil communities. Ideally, species means should be derived from comparable study numbers, which was not achieved here. These steps would improve SSD reliability, although data availability is still a challenge.

Overlaying the SSD and EED curves graphically is an effective way of visualizing risk. The inclusion of MC2D simulations and calculation of the HC5 (percentile approach) offers a complementary and robust method of probabilistic RA. The MC2D method is a statistically robust simulation, while the HC_5_ has the advantage of extracting a specific toxicity threshold. Future studies could adopt these methods, enhanced by better field data, refined thresholds, and improved species representation in SSDs, subject to the availability of sufficient data.

This research enhances our understanding of the risks associated with MPs in the context of sewage sludge land application. It suggests that highly MP-contaminated sludge samples should be diverted to alternative disposal methods, while less-contaminated sludge may still offer agronomic benefits when applied to land due to its high nutrient content. A more targeted and adaptive management approach could help keep MP levels in soil within acceptable limits for soil biota, as defined quantitatively in this study, thereby contributing to the preservation of soil ecosystems.

Protecting ecosystem health is essential for both the environment and human society, as humans are deeply interconnected with the ecosystems on which they depend on. Ensuring the safety of soil biota is especially important in agricultural contexts, as it supports the production of healthy crops. Some of these crops may be consumed by livestock**—**potentially leading to human food products**—**or, under certain conditions and treatments, be consumed directly by humans. Ultimately, promoting a healthier soil ecosystem is a valuable way to contribute to a healthier society.

## Supplementary Material



## Notes

*R* code and output; *R* code for reproducibility in the *R* software directly; data for MPs in SAS; data for MPs in sludge; data for effect thresholds (NOEC_equivalents_); and further information supporting the present study (ZIP)

## List of Abbreviations

AIC: Akaike information criterion
CDF: cumulative distribution function
dw: dry weight
EED: environmental exposure distribution
HC5: hazard concentration inducing effects on 5% of species
HONEC: highest observed no effect concentration
LOEC: lowest observed effect concentration
MC2D: two-dimensional Monte Carlo simulation
nb/kg dw: number per kilogram dry weight
NOEC: no observed effect concentration
PNEC: predicted no effect concentration
pyr-GCMS: pyrolysis-gas chromatography–mass spectrometry
RA: risk assessment
ref: reference
SAS: sludge-amended soils
SI: Supporting Information
SSD: species-sensitivity distribution
TGA: thermogravimetric analysis
UF: uncertainty factor
w/w: weight/weight
WWTP: wastewater treatment plant
