# Supplementary material for: A Quantitative Environmental Risk Assessment for Microplastics in Sewage Sludge Applied to Land
Source: Environ Sci Technol. 2025 Oct 20;59(49):26526–38. doi: 10.1021/acs.est.5c08026 (PMC12713786; doi:10.1021/acs.est.5c08026)
Supplement: Supplementary file 1 [file es5c08026_si_001.zip › 2_Supporting information.docx]

SUPPORTING INFORMATION

Paul Boisseaux, Marie Laure Delignette-Muller, Tamara Galloway. *A quantitative environmental risk assessment for microplastics in sewage sludge applied to land*. **2025**.

Table of Figures

[Figure_S 1: Diagram for selecting toxicological values and uncertainty factors 5](#Figure_S!0|sequence)

Index of Tables

Table_S 1: Further criteria for SSD’ study inclusion or exclusion 1

Table_S 2: Selection of sensitivity thresholds for building the SSD 1

Table_S 3: Density of polymers used for mass to number concentration conversions 2

Table_S 4: Uncertainty factors used for the calculation of NOEC-equivalent 4

Table_S 5: Studies retained for control soils and SAS 5

Table_S 1: Further criteria for SSD’ study inclusion or exclusion

| Additional exclusion criteria were applied, resulting in more stringent standards to enhance data quality. Short-term studies (< 5 days) were excluded as we focused only on subchronic or chronic studies. Bioplastics and nanoplastics (items < 1 µm) were excluded, so only items in the range 1 µm – 5000 µm were included. One exception was made for “long fibres” (mean length 11.9 mm, diameter 7.5 µm) (Selonen et al., 2020). Studies that expressed MP concentrations as weight per dry weight either in percentage or absolute value (e.g., % w/w or g/kg dw) or number per dry weight (e.g., nb/kg dw) were included. Studies that expressed MPs concentrations in other units were excluded (e.g., nb/m2). |
| --- |

Table_S 2: Selection of sensitivity thresholds for building the SSD

| Because there was a wide array of studies in different branches of ecotoxicology and ecology, on a wide array of species, which employ different approaches and tackle complex questions not limited to providing toxicological values, it was impossible to have a strict and homogenous statistical criteria approach.  In the majority of studies, the determination of a sensitivity threshold was based on statistical significance or models, according to the employed method such as one-way ANOVA, post-hoc tests (Dunnett, Tukey, Duncan, LSD), T-tests, non-parametric tests (Kruskal-Wallis, Mann-Whitney), Fisher's exact test, Spearman rank correlation, effect concentration (ECx) modelling, or Bayesian approaches. Comparisons were done with the controls with a *p* threshold of 0.05 or 75% for Bayesian approach as described in studies. In some cases, where the lack of statistical post-hoc analysis shed the doubt on the significance of effects, those endpoints were excluded. In one instance, supplementary data was re-analysed with an additional statistical test and the data was included. The EC_10_ or LC_10_ (lethal concentration for 10% of the population) was considered extractable as a sensitivity threshold, so as a LOEC, but this occurred in only one article.  In a few studies, the significant difference to extract the sensitivity threshold was determined based on the overall interpretation of the study's elements rather than solely on statistical p-value significance. This included one case where a significant Kruskal-Wallis test result was reported without a post-hoc test but where the graph and text indicated a strong effect starting from a particular concentration, mentions of significant effects at specific concentrations in the text, overall article emphasis and expert judgment e.g. with clear effects in PCA (Principal Component Analysis). |
| --- |

Table_S 3: Density of polymers used for mass to number concentration conversions

| abbreviation | value | ref |
| --- | --- | --- |
| Epoxy resin | 0.905 | As in SM of Amorim et. al. 2021: KSR4525, Borealis AG, Vienna, Austria thermosetting Bisphenol-A resin  <https://www.borealisgroup.com/products/product-catalogue/ksr4525-1>  Density = 905 kg/m³ |
| PP | 0.91 | 0.91 (SOURCE https://plasticseurope.org/plastics-explained/a-large-family/polyolefins/) |
| LDPE | 0.92 | 0.92 (SOURCE: https://plasticseurope.org/plastics-explained/a-large-family/polyolefins/) |
| PE | 0.93 | 0.93 (average of all possible kind of PE, source: https://omnexus.specialchem.com/selection-guide/polyethylene-plastic) |
| HDPE | 0.95 | 0.95 (https://plasticseurope.org/plastics-explained/a-large-family/polyolefins/) |
| PE (Ren et al 2020) | 0.95 |  |
| PE (Ju et al 2019) | 0.96 |  |
| PS | 1 | 1.00 (source: https://plasticranger.com/density-of-polystyrene/) |
| Nylon/PA | 1.15 | 1.15 (source: http://laminatedplastics.com/nylon) |
| acrylic | 1.185 | https://www.matweb.com/search/datasheet.aspx?bassnum=O1303&ckck=1 |
| PC | 1.2 | 1.20 (source: https://www.bpf.co.uk/plastipedia/polymers/Polycarbonate.aspx) |
| tire | 1.2 | 1.2 (source https://doi.org/10.1016/j.chemosphere.2019.01.176 ) |
| PAN (PAN - Polyacrylonitrile ) | 1.125 | 1.125 (source https://omnexus.specialchem.com/polymer-property/densit |
| PET | 1.38 | assumed PET: 1.38 (source https://scipoly.com/density-of-polymers-by-density/) |
| PES (polyester) | 1.38 | assumed PET: 1.38 (source https://scipoly.com/density-of-polymers-by-density/ and also https://vnpolyfiber.com/basic-information-of-polyester-staple-fiber/#:~:text=Physical%20properties%20of%20Polyester%20Fibers%3A&text=Density%20%3A%201.39%20g%2Fcc,40%20to%2080%20cN%2Ftex )1.39 of Polyester Fibers in |
| PVC | 1.38 | 1.38 (source: https://www.bpf.co.uk/plastipedia/polymers/PVC.aspx) |
| EPDM | 1.6 | 1.6 (van Kleunen et al. (2019)) |
|  |  |  |

Table_S 4: Uncertainty factors used for the calculation of NOEC-equivalent

| **Time** | **Description** | **UF_T_** |
| --- | --- | --- |
| Chronic 2 | >41d | 1 |
| Chronic 1 | 21d-41d | 2 |
| Subchronic | 5-20d | 4 |
| Acute | 0-5d | - |
| **Descriptor precision and extent of effect** | **Description** | **UF_E_** |
| NOEC_a | Good quality, dose-response | 1 |
| NOEC_b or HONEC | Alternative | 1 |
| Sensitivity threshold LOEC_a | Good quality, dose-response | 2 |
| Sensitivity threshold  LOEC_b | Alternative | 4 |
| **Endpoint category** | **Description** | **UF_C_** |
| Type A | e.g., Enzymatic activities, transcriptomics/genomics,  bacterial diversity, energy reserves, etc. | 1 |
| Type B | Reproduction/germination/growth/morphological traits | 2 |
| Type C | Survival | 4 |


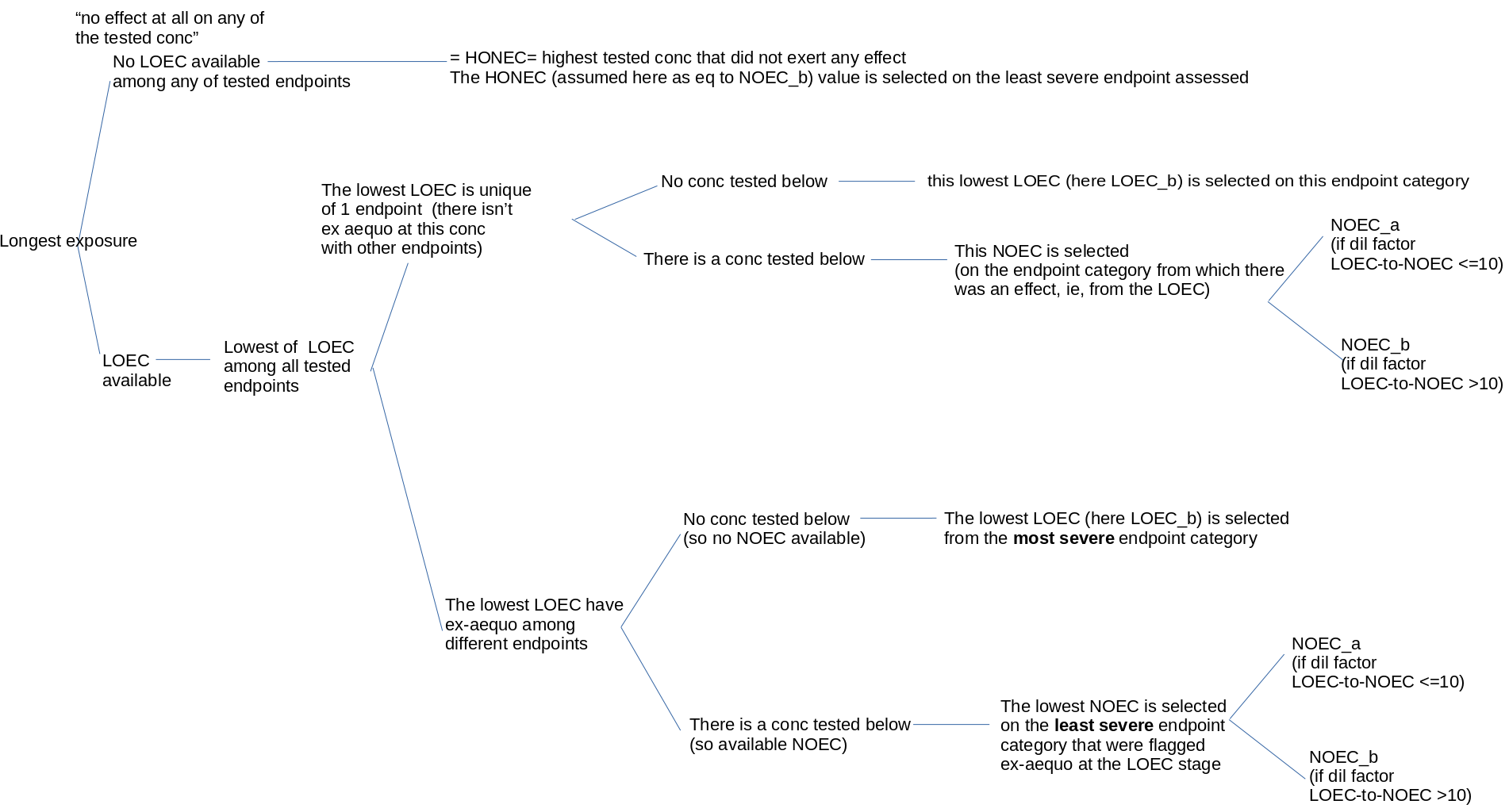
Figure_S 1: Diagram for selecting toxicological values and uncertainty factors

**References for retained studies on microplastics in soil-amended sludge and control soils**

Table_S 5: Studies retained for control soils and SAS

|  |  | Ctrl | SAS |
| --- | --- | --- | --- |
| 1 | Adhikari2024 | X | X |
| 2 | Amirhosseni 2023 | X | X |
| 3 | Beni2023 | X | X |
| 4 | Corradini 2019 | X | X |
| 5 | Crossman 2020 | X | X |
| 6 | Heerey 2023 | X | X |
| 7 | Klemmensen2024 |  | X |
| 8 | Piehl2018 | X |  |
| 9 | Radford 2023 | X | X |
| 10 | Ragoobur 2021 | X |  |
| 11 | Schell 2022 | X | X |
| 12 | Tagg 2022 | X | X |
| 13 | Tran 2023 | X | X |
| 14 | van den Berg 2020 | X | X |
| 15 | Yang2021 | X | X |
| 16 | Yi2023 | X | X |
| 17 | Zhang and Liu 2018 |  | X |
| 18 | Zhang2020 | X | X |
| 19 | Zubris 2005 | X | X |

Adhikari, K., Pearce, C.I., Sanguinet, K.A., Bary, A.I., Chowdhury, I., Eggleston, I., Xing, B., Flury, M., 2024. Accumulation of microplastics in soil after long-term application of biosolids and atmospheric deposition. Science of the Total Environment 912, 168883.

Amirhosseini, K., Haghani, Z., Alikhani, H.A., 2023. Microplastics pollution in rice fields: A case study of Pir Bazar rural district of Gilan, Iran. Environmental Monitoring and Assessment 195, 1473.

Corradini, F., Meza, P., Eguiluz, R., Casado, F., Huerta-Lwanga, E., Geissen, V., 2019. Evidence of microplastic accumulation in agricultural soils from sewage sludge disposal. Science of the total environment 671, 411–420.

Crossman, J., Hurley, R.R., Futter, M., Nizzetto, L., 2020. Transfer and transport of microplastics from biosolids to agricultural soils and the wider environment. Science of the Total Environment 724, 138334.

Heerey, L., O’Sullivan, J.J., Bruen, M., Turner, J., Mahon, A.M., Murphy, S., Lally, H.T., O’Connor, J.D., O’Connor, I., Nash, R., 2023. Export pathways of biosolid derived microplastics in soil systems – Findings from a temperate maritime climate. Science of The Total Environment 888, 164028. https://doi.org/10.1016/j.scitotenv.2023.164028

Klemmensen, N.D.R., Chand, R., Blanco, M.S., Vollertsen, J., 2024. Microplastic abundance in sludge-treated fields: Variance and estimated half-life. Science of The Total Environment 922, 171394. https://doi.org/10.1016/j.scitotenv.2024.171394

Naderi Beni, N., Karimifard, S., Gilley, J., Messer, T., Schmidt, A., Bartelt-Hunt, S., 2023. Higher concentrations of microplastics in runoff from biosolid-amended croplands than manure-amended croplands. Communications Earth & Environment 4, 42.

Piehl, S., Leibner, A., Löder, M.G.J., Dris, R., Bogner, C., Laforsch, C., 2018. Identification and quantification of macro- and microplastics on an agricultural farmland. Scientific Reports 8, 17950. https://doi.org/10.1038/s41598-018-36172-y

Radford, F., Horton, A., Hudson, M., Shaw, P., Williams, I., 2023. Agricultural soils and microplastics: Are biosolids the problem? Frontiers in Soil Science 2, 941837.

Ragoobur, D., Huerta-Lwanga, E., Somaroo, G.D., 2021. Microplastics in agricultural soils, wastewater effluents and sewage sludge in Mauritius. Science of The Total Environment 798, 149326. https://doi.org/10.1016/j.scitotenv.2021.149326

Schell, T., Hurley, R., Buenaventura, N.T., Mauri, P.V., Nizzetto, L., Rico, A., Vighi, M., 2022. Fate of microplastics in agricultural soils amended with sewage sludge: Is surface water runoff a relevant environmental pathway? Environmental Pollution 293, 118520.

Tagg, A.S., Brandes, E., Fischer, F., Fischer, D., Brandt, J., Labrenz, M., 2022. Agricultural application of microplastic-rich sewage sludge leads to further uncontrolled contamination. Science of The Total Environment 806, 150611. https://doi.org/10.1016/j.scitotenv.2021.150611

Tran, T.K.A., Raju, S., Singh, A., Senathirajah, K., Bhagwat-Russell, G., Daggubati, L., Kandaiah, R., Palanisami, T., 2023. Occurrence and distribution of microplastics in long-term biosolid-applied rehabilitation land: An overlooked pathway for microplastic entry into terrestrial ecosystems in Australia. Environmental Pollution 336, 122464. https://doi.org/10.1016/j.envpol.2023.122464

van den Berg, P., Huerta-Lwanga, E., Corradini, F., Geissen, V., 2020. Sewage sludge application as a vehicle for microplastics in eastern Spanish agricultural soils. Environmental Pollution 261, 114198. https://doi.org/10.1016/j.envpol.2020.114198

Yang, J., Li, L., Li, R., Xu, L., Shen, Y., Li, S., Tu, C., Wu, L., Christie, P., Luo, Y., 2021. Microplastics in an agricultural soil following repeated application of three types of sewage sludge: A field study. Environmental Pollution 289, 117943. https://doi.org/10.1016/j.envpol.2021.117943

Yi, S., Zuo, W., Xu, L., Wang, Y., Gu, C., Shan, Y., Bai, Y., 2023. Accumulation and migration of microplastics and its influencing factors in coastal saline-alkali soils amended with sewage sludge. Ecotoxicology and Environmental Safety 266, 115597. https://doi.org/10.1016/j.ecoenv.2023.115597

Zhang, G.S., Liu, Y.F., 2018. The distribution of microplastics in soil aggregate fractions in southwestern China. Science of The Total Environment 642, 12–20. https://doi.org/10.1016/j.scitotenv.2018.06.004

Zhang, L., Xie, Y., Liu, J., Zhong, S., Qian, Y., Gao, P., 2020. An Overlooked Entry Pathway of Microplastics into Agricultural Soils from Application of Sludge-Based Fertilizers. Environ. Sci. Technol. 54, 4248–4255. https://doi.org/10.1021/acs.est.9b07905

Zubris, K.A.V., Richards, B.K., 2005. Synthetic fibers as an indicator of land application of sludge. Environmental Pollution 138, 201–211. https://doi.org/10.1016/j.envpol.2005.04.013

**References for retained sewage sludge studies**

Chand, R., Rasmussen, L.A., Tumlin, S., Vollertsen, J., 2021. The occurrence and fate of microplastics in a mesophilic anaerobic digester receiving sewage sludge, grease, and fatty slurries. Science of the Total Environment 798, 149287.

Chen, Z., Zhao, W., Xing, R., Xie, S., Yang, X., Cui, P., Lü, J., Liao, H., Yu, Z., Wang, S., 2020. Enhanced in situ biodegradation of microplastics in sewage sludge using hyperthermophilic composting technology. Journal of hazardous materials 384, 121271.

Corradini, F., Meza, P., Eguiluz, R., Casado, F., Huerta-Lwanga, E., Geissen, V., 2019. Evidence of microplastic accumulation in agricultural soils from sewage sludge disposal. Science of the total environment 671, 411–420.

Crossman, J., Hurley, R.R., Futter, M., Nizzetto, L., 2020. Transfer and transport of microplastics from biosolids to agricultural soils and the wider environment. Science of the Total Environment 724, 138334.

Edo, C., González-Pleiter, M., Leganés, F., Fernández-Piñas, F., Rosal, R., 2020. Fate of microplastics in wastewater treatment plants and their environmental dispersion with effluent and sludge. Environmental Pollution 259, 113837.

El Hayany, B., El Fels, L., Quénéa, K., Dignac, M.-F., Rumpel, C., Gupta, V.K., Hafidi, M., 2020. Microplastics from lagooning sludge to composts as revealed by fluorescent staining-image analysis, Raman spectroscopy and pyrolysis-GC/MS. Journal of Environmental Management 275, 111249.

Harley-Nyang, D., Memon, F.A., Jones, N., Galloway, T., 2022. Investigation and analysis of microplastics in sewage sludge and biosolids: A case study from one wastewater treatment works in the UK. Science of the Total Environment 823, 153735.

Hernández-Arenas, R., Beltrán-Sanahuja, A., Navarro-Quirant, P., Sanz-Lazaro, C., 2021. The effect of sewage sludge containing microplastics on growth and fruit development of tomato plants. Environmental Pollution 268, 115779.

Horton, A.A., Cross, R.K., Read, D.S., Jürgens, M.D., Ball, H.L., Svendsen, C., Vollertsen, J., Johnson, A.C., 2021. Semi-automated analysis of microplastics in complex wastewater samples. Environmental Pollution 268, 115841.

Lares, M., Ncibi, M.C., Sillanpää, Markus, Sillanpää, Mika, 2018. Occurrence, identification and removal of microplastic particles and fibers in conventional activated sludge process and advanced MBR technology. Water research 133, 236–246.

Li, X., Chen, L., Mei, Q., Dong, B., Dai, X., Ding, G., Zeng, E.Y., 2018. Microplastics in sewage sludge from the wastewater treatment plants in China. Water Research 142, 75–85. https://doi.org/10.1016/j.watres.2018.05.034

Li, X., Liu, H., Wang, L., Guo, H., Zhang, J., Gao, D., 2022. Effects of typical sludge treatment on microplastics in China—characteristics, abundance and micro-morphological evidence. Science of the Total Environment 826, 154206.

Lusher, A.L., Hurley, R., Vogelsang, C., Nizzetto, L., Olsen, M., 2017. Mapping microplastics in sludge.

Mahon, A.M., O’Connell, B., Healy, M.G., O’Connor, I., Officer, R., Nash, R., Morrison, L., 2017. Microplastics in sewage sludge: effects of treatment. Environmental Science & Technology 51, 810–818.

Petroody, S.S.A., Hashemi, S.H., van Gestel, C.A., 2021. Transport and accumulation of microplastics through wastewater treatment sludge processes. Chemosphere 278, 130471.

Rasmussen, L.A., Iordachescu, L., Tumlin, S., Vollertsen, J., 2021. A complete mass balance for plastics in a wastewater treatment plant-macroplastics contributes more than microplastics. Water Research 201, 117307.

Salmi, P., Ryymin, K., Karjalainen, A.K., Mikola, A., Uurasjärvi, E., Talvitie, J., 2021. Particle balance and return loops for microplastics in a tertiary-level wastewater treatment plant. Water Science and Technology 84, 89–100.

Schell, T., Hurley, R., Buenaventura, N.T., Mauri, P.V., Nizzetto, L., Rico, A., Vighi, M., 2022. Fate of microplastics in agricultural soils amended with sewage sludge: Is surface water runoff a relevant environmental pathway? Environmental Pollution 293, 118520.

Tagg, A.S., Brandes, E., Fischer, F., Fischer, D., Brandt, J., Labrenz, M., 2022. Agricultural application of microplastic-rich sewage sludge leads to further uncontrolled contamination. Science of The Total Environment 806, 150611. https://doi.org/10.1016/j.scitotenv.2021.150611

Wiśniowska, E., Moraczewska-Majkut, K., Nocoń, W., 2018. Efficiency of microplastics removal in selected wastewater treatment plants-preliminary studies.

Xu, Q., Gao, Y., Xu, L., Shi, W., Wang, F., LeBlanc, G.A., Cui, S., An, L., Lei, K., 2020. Investigation of the microplastics profile in sludge from China’s largest Water reclamation plant using a feasible isolation device. Journal of hazardous materials 388, 122067.

Yang, Z., Li, S., Ma, S., Liu, P., Peng, D., Ouyang, Z., Guo, X., 2021. Characteristics and removal efficiency of microplastics in sewage treatment plant of Xi’an City, northwest China. Science of the Total Environment 771, 145377.

Yuan, F., Zhao, H., Sun, H., Sun, Y., Zhao, J., Xia, T., 2022. Investigation of microplastics in sludge from five wastewater treatment plants in Nanjing, China. Journal of Environmental Management 301, 113793.

Zhang, Yunhai, Wang, H., Xu, J., Su, X., Lu, M., Wang, Z., Zhang, Yongjun, 2021. Occurrence and characteristics of microplastics in a wastewater treatment plant. Bulletin of Environmental Contamination and Toxicology 1–7.

Ziajahromi, S., Neale, P.A., Silveira, I.T., Chua, A., Leusch, F.D., 2021. An audit of microplastic abundance throughout three Australian wastewater treatment plants. Chemosphere 263, 128294.

**References for studies retained in the SSD**

Amorim, M.J., Scott-Fordsmand, J.J., 2021. Plastic pollution–a case study with Enchytraeus crypticus–from micro-to nanoplastics. Environmental Pollution 271, 116363.

Boots, B., Russell, C.W., Green, D.S., 2019. Effects of Microplastics in Soil Ecosystems: Above and Below Ground. Environ. Sci. Technol. 53, 11496–11506. https://doi.org/10.1021/acs.est.9b03304

Cao, D., Wang, X., Luo, X., Liu, G., Zheng, H., 2017. Effects of polystyrene microplastics on the fitness of earthworms in an agricultural soil, in: IOP Conference Series: Earth and Environmental Science. IOP Publishing, p. 012148.

Chen, Y., Liu, X., Leng, Y., Wang, J., 2020. Defense responses in earthworms (Eisenia fetida) exposed to low-density polyethylene microplastics in soils. Ecotoxicology and Environmental Safety 187, 109788. https://doi.org/10.1016/j.ecoenv.2019.109788

Cheng, Y., Song, W., Tian, H., Zhang, K., Li, B., Du, Z., Zhang, W., Wang, Jinhua, Wang, Jun, Zhu, L., 2021. The effects of high-density polyethylene and polypropylene microplastics on the soil and earthworm Metaphire guillelmi gut microbiota. Chemosphere 267, 129219. https://doi.org/10.1016/j.chemosphere.2020.129219

de Souza Machado, A.A., Lau, C.W., Kloas, W., Bergmann, J., Bachelier, J.B., Faltin, E., Becker, R., Görlich, A.S., Rillig, M.C., 2019. Microplastics Can Change Soil Properties and Affect Plant Performance. Environ. Sci. Technol. 53, 6044–6052. https://doi.org/10.1021/acs.est.9b01339

Ding, J., Zhu, D., Wang, H.-T., Lassen, S.B., Chen, Q.-L., Li, G., Lv, M., Zhu, Y.-G., 2020. Dysbiosis in the Gut Microbiota of Soil Fauna Explains the Toxicity of Tire Tread Particles. Environ. Sci. Technol. 54, 7450–7460. https://doi.org/10.1021/acs.est.0c00917

Fei, Y., Huang, S., Zhang, H., Tong, Y., Wen, D., Xia, X., Wang, H., Luo, Y., Barceló, D., 2020. Response of soil enzyme activities and bacterial communities to the accumulation of microplastics in an acid cropped soil. Science of The Total Environment 707, 135634. https://doi.org/10.1016/j.scitotenv.2019.135634

Huang, Y., Zhao, Y., Wang, J., Zhang, M., Jia, W., Qin, X., 2019. LDPE microplastic films alter microbial community composition and enzymatic activities in soil. Environmental Pollution 254, 112983. https://doi.org/10.1016/j.envpol.2019.112983

Huerta Lwanga, E., Gertsen, H., Gooren, H., Peters, P., Salánki, T., van der Ploeg, M., Besseling, E., Koelmans, A.A., Geissen, V., 2016. Microplastics in the Terrestrial Ecosystem: Implications for Lumbricus terrestris (Oligochaeta, Lumbricidae). Environ. Sci. Technol. 50, 2685–2691. https://doi.org/10.1021/acs.est.5b05478

Jiang, X., Chang, Y., Zhang, T., Qiao, Y., Klobučar, G., Li, M., 2020. Toxicological effects of polystyrene microplastics on earthworm (Eisenia fetida). Environmental Pollution 259, 113896. https://doi.org/10.1016/j.envpol.2019.113896

Ju, H., Zhu, D., Qiao, M., 2019. Effects of polyethylene microplastics on the gut microbial community, reproduction and avoidance behaviors of the soil springtail, Folsomia candida. Environmental Pollution 247, 890–897. https://doi.org/10.1016/j.envpol.2019.01.097

Lahive, E., Walton, A., Horton, A.A., Spurgeon, D.J., Svendsen, C., 2019. Microplastic particles reduce reproduction in the terrestrial worm Enchytraeus crypticus in a soil exposure. Environmental Pollution 255, 113174. https://doi.org/10.1016/j.envpol.2019.113174

Li, B., Song, W., Cheng, Y., Zhang, K., Tian, H., Du, Z., Wang, Jinhua, Wang, Jun, Zhang, W., Zhu, L., 2021. Ecotoxicological effects of different size ranges of industrial-grade polyethylene and polypropylene microplastics on earthworms Eisenia fetida. Science of The Total Environment 783, 147007. https://doi.org/10.1016/j.scitotenv.2021.147007

Li, Z., Li, Q., Li, R., Zhao, Y., Geng, J., Wang, G., 2020. Physiological responses of lettuce (Lactuca sativa L.) to microplastic pollution. Environmental Science and Pollution Research 27, 30306–30314. https://doi.org/10.1007/s11356-020-09349-0

Liang, Y., Lehmann, A., Ballhausen, M.-B., Muller, L., Rillig, M.C., 2019. Increasing temperature and microplastic fibers jointly influence soil aggregation by saprobic fungi. Frontiers in Microbiology 10, 2018.

Ma, J., Sheng, G.D., O’Connor, P., 2020. Microplastics combined with tetracycline in soils facilitate the formation of antibiotic resistance in the Enchytraeus crypticus microbiome. Environmental Pollution 264, 114689. https://doi.org/10.1016/j.envpol.2020.114689

Pflugmacher, S., Sulek, A., Mader, H., Heo, J., Noh, J.H., Penttinen, O.-P., Kim, Y., Kim, S., Esterhuizen, M., 2020. The Influence of New and Artificial Aged Microplastic and Leachates on the Germination of Lepidium sativum L. Plants.

Pignattelli, S., Broccoli, A., Piccardo, M., Felline, S., Terlizzi, A., Renzi, M., 2021. Short-term physiological and biometrical responses of Lepidium sativum seedlings exposed to PET-made microplastics and acid rain. Ecotoxicology and Environmental Safety 208, 111718. https://doi.org/10.1016/j.ecoenv.2020.111718

Pignattelli, S., Broccoli, A., Renzi, M., 2020. Physiological responses of garden cress (L. sativum) to different types of microplastics. Science of The Total Environment 727, 138609. https://doi.org/10.1016/j.scitotenv.2020.138609

Prendergast-Miller, M.T., Katsiamides, A., Abbass, M., Sturzenbaum, S.R., Thorpe, K.L., Hodson, M.E., 2019. Polyester-derived microfibre impacts on the soil-dwelling earthworm Lumbricus terrestris. Environmental Pollution 251, 453–459. https://doi.org/10.1016/j.envpol.2019.05.037

Qi, Y., Yang, X., Pelaez, A.M., Huerta Lwanga, E., Beriot, N., Gertsen, H., Garbeva, P., Geissen, V., 2018. Macro- and micro- plastics in soil-plant system: Effects of plastic mulch film residues on wheat (Triticum aestivum) growth. Science of The Total Environment 645, 1048–1056. https://doi.org/10.1016/j.scitotenv.2018.07.229

Ren, X., Tang, J., Liu, X., Liu, Q., 2020. Effects of microplastics on greenhouse gas emissions and the microbial community in fertilized soil. Environmental Pollution 256, 113347. https://doi.org/10.1016/j.envpol.2019.113347

Rodríguez-Seijo, A., da Costa, J.P., Rocha-Santos, T., Duarte, A.C., Pereira, R., 2018. Oxidative stress, energy metabolism and molecular responses of earthworms (Eisenia fetida) exposed to low-density polyethylene microplastics. Environmental Science and Pollution Research 25, 33599–33610. https://doi.org/10.1007/s11356-018-3317-z

Rodriguez-Seijo, A., Lourenço, J., Rocha-Santos, T.A.P., da Costa, J., Duarte, A.C., Vala, H., Pereira, R., 2017. Histopathological and molecular effects of microplastics in Eisenia andrei Bouché. Environmental Pollution 220, 495–503. https://doi.org/10.1016/j.envpol.2016.09.092

Selonen, S., Dolar, A., Jemec Kokalj, A., Skalar, T., Parramon Dolcet, L., Hurley, R., van Gestel, C.A.M., 2020. Exploring the impacts of plastics in soil – The effects of polyester textile fibers on soil invertebrates. Science of The Total Environment 700, 134451. https://doi.org/10.1016/j.scitotenv.2019.134451

Sobhani, Z., Fang, C., Naidu, R., Megharaj, M., 2021a. Microplastics as a vector of toxic chemicals in soil: Enhanced uptake of perfluorooctane sulfonate and perfluorooctanoic acid by earthworms through sorption and reproductive toxicity. Environmental Technology & Innovation 22, 101476. https://doi.org/10.1016/j.eti.2021.101476

Sobhani, Z., Panneerselvan, L., Fang, C., Naidu, R., Megharaj, M., 2021b. Chronic and Transgenerational Effects of Polystyrene Microplastics at Environmentally Relevant Concentrations in Earthworms (Eisenia fetida). Environmental Toxicology and Chemistry 40, 2240–2246. https://doi.org/10.1002/etc.5072

Song, Y., Cao, C., Qiu, R., Hu, J., Liu, M., Lu, S., Shi, H., Raley-Susman, K.M., He, D., 2019. Uptake and adverse effects of polyethylene terephthalate microplastics fibers on terrestrial snails (Achatina fulica) after soil exposure. Environmental Pollution 250, 447–455. https://doi.org/10.1016/j.envpol.2019.04.066

van Kleunen, M., Brumer, A., Gutbrod, L., Zhang, Z., 2020. A microplastic used as infill material in artificial sport turfs reduces plant growth. Plants, people, planet 2, 157–166.

Wang, F., Zhang, X., Zhang, Shuqi, Zhang, Shuwu, Sun, Y., 2020. Interactions of microplastics and cadmium on plant growth and arbuscular mycorrhizal fungal communities in an agricultural soil. Chemosphere 254, 126791.

Wang, J., Coffin, S., Sun, C., Schlenk, D., Gan, J., 2019. Negligible effects of microplastics on animal fitness and HOC bioaccumulation in earthworm Eisenia fetida in soil. Environmental Pollution 249, 776–784. https://doi.org/10.1016/j.envpol.2019.03.102

Xu, G., Yu, Y., 2021. Polystyrene microplastics impact the occurrence of antibiotic resistance genes in earthworms by size-dependent toxic effects. Journal of Hazardous Materials 416, 125847.

Yan, Y., Chen, Z., Zhu, F., Zhu, C., Wang, C., Gu, C., 2021. Effect of Polyvinyl Chloride Microplastics on Bacterial Community and Nutrient Status in Two Agricultural Soils. Bulletin of Environmental Contamination and Toxicology 107, 602–609. https://doi.org/10.1007/s00128-020-02900-2

Yi, M., Zhou, S., Zhang, L., Ding, S., 2021. The effects of three different microplastics on enzyme activities and microbial communities in soil. Water Environment Research 93, 24–32. https://doi.org/10.1002/wer.1327

Zhou, Y., Liu, X., Wang, J., 2020. Ecotoxicological effects of microplastics and cadmium on the earthworm Eisenia foetida. Journal of Hazardous Materials 392, 122273. https://doi.org/10.1016/j.jhazmat.2020.122273

Zhu, D., Chen, Q.-L., An, X.-L., Yang, X.-R., Christie, P., Ke, X., Wu, L.-H., Zhu, Y.-G., 2018. Exposure of soil collembolans to microplastics perturbs their gut microbiota and alters their isotopic composition. Soil Biology and Biochemistry 116, 302–310. <https://doi.org/10.1016/j.soilbio.2017.10.027>
