# Supplementary material for: A Quantitative Environmental Risk Assessment for Microplastics in Sewage Sludge Applied to Land
Source: Environ Sci Technol. 2025 Oct 20;59(49):26526–38. doi: 10.1021/acs.est.5c08026 (PMC12713786; doi:10.1021/acs.est.5c08026)
Supplement: Supplementary file 1 [file es5c08026_si_001.zip › Cover sheet for SI files.docx]

**Summary of supporting information files:**

| Name | Quick description | Number of pages |
| --- | --- | --- |
| “SSD_and_EED_fit_article.pdf” | This document contains the *R* code used for the statistical analysis presented in the paper. It includes code for fitting SSD and EED models, as well as for running MC2D simulations, along with the output of each command, all compiled in PDF format. The document is organized into clearly defined sections for step-by-step reading, includes a table of contents at the beginning, and features comments throughout to aid understanding. | 26 |
| “SSD_and_EED_fit.Rmd” | R Markdown (.RMD) file containing code for fitting SSD and EED models and running MC2D simulations. Includes annotated steps previously compiled in PDF format. | 1 |
| “MPs_in_sludge_amended_soils_light.csv” | CSV data file containing concentrations of MPs in SAS and control soil samples. | 1 |
| “sludge_conc_light.csv” | CSV data file containing concentrations of MPs in stabilized sewage sludge, representing a stage at which it may potentially be applied to land. | 1 |
| “SSD_dataset_simplified_r.csv” | A CSV-format dataset presenting the NOEC_equivalent_ values of MPs for various soil species, used in the construction of the SSD. The dataset includes descriptors, applied UFs, and characteristics of the MPs. | 1 |
| “2_Supporting information.docx” | This document contains additional supporting information for the study, provided in .docx format. | 11 |
