# Supplementary material for: A Quantitative Environmental Risk Assessment for Microplastics in Sewage Sludge Applied to Land
Source: Environ Sci Technol. 2025 Oct 20;59(49):26526–38. doi: 10.1021/acs.est.5c08026 (PMC12713786; doi:10.1021/acs.est.5c08026)
Supplement: Supplementary file 1 [file es5c08026_si_001.zip › SSD_and_EED_fit_article.pdf]

Environmental Exposure Distributions (EED) of MPs in sludge and sludge-amended soils

and

Species sensitivity distribution (SSD) of MPs to soils species

and MC2D simulations from those distributions*

Paul Boisseaux, Marie-Laure Delignette-Muller, Tamara Galloway

2025-02-19

```
### abbreviation: MPs: microplastics
### abbreviation: SSD: Species sensitivity distributions
### abbreviation: EED: Environmental Exposure Distributions
### abbreviation: SAS: sludge-amended soils
### abbreviation: MC2D: Two dimensional Monte carlo simulations
```

*Research article: "A quantitative environmental risk assessment for microplastics in sewage sludge applied to land"

Contents

1 EED **3**
1.1 Boxplots of Control soils and SAS . . . 3
1.2 EED of SAS: model fits (Burr, log-normal, log-logistic, weibull, gamma) . . . 5
1.3 EED of Control soils: model fits (Burr, log-normal, log-logistic, weibull, gamma) . . . 8
1.4 EED sludge (Burr, log-normal, log-logistic, weibull, gamma) . . . 11
1.4.1 Undiluted concentrations (treated/stabilised sludge that could potentially be applied to lands) . . . 11

2 SSD on soil species (log-normal, log-logistic and Burr) **14**
2.1 Averaged by species . . . 14

3 Monte Carlo simulations for different sludge dilution factors **16**
3.1 Uncertainty dimension . . . 16
3.2 Code to simulate variability conditionally to uncertainty (on 1 scenario) . . . 16
3.3 Repeats for 17 dilution scenarios . . . 18

4 Monte Carlo simulations for control soils **22**

5 Monte Carlo simulations for SAS **24**

```r
# Loading of required packages
library(fitdistrplus)
library(mc2d)
library(actuar)
library(ggplot2)
library(dplyr)
```

1 EED

1.1 Boxplots of Control soils and SAS

```r
# Definition of the seed of the random number generator
# to make the results reproducible
set.seed(123)
a <- read.csv("MPs_in_sludge_amended_soils_light.csv",
              header = TRUE, row.names = NULL, stringsAsFactors = TRUE)
# conc are in nb/kg dw

a$conc_nb_g <- a$conc/1000 # we change to nb/g
# this is because smaller numerical values are better handled in R

# Modification of names of cat modalities
levels(a$cat) <- c("SAS", "ctrl")
boxplot(conc_nb_g ~ cat, data = a)
```

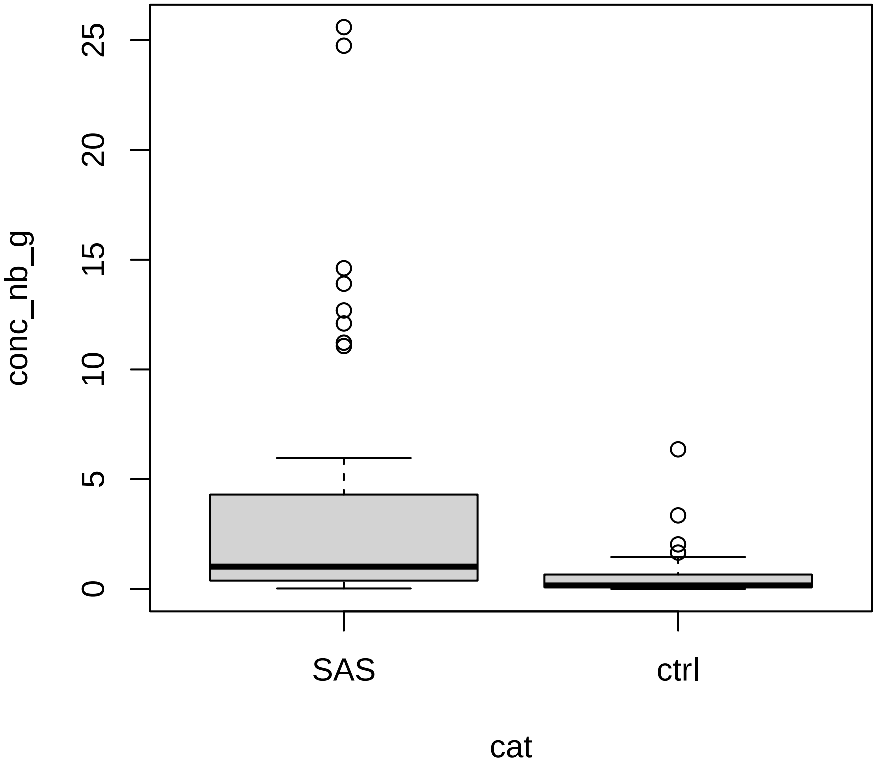

```r
boxplot(log(conc_nb_g) ~ cat, data = a)
```

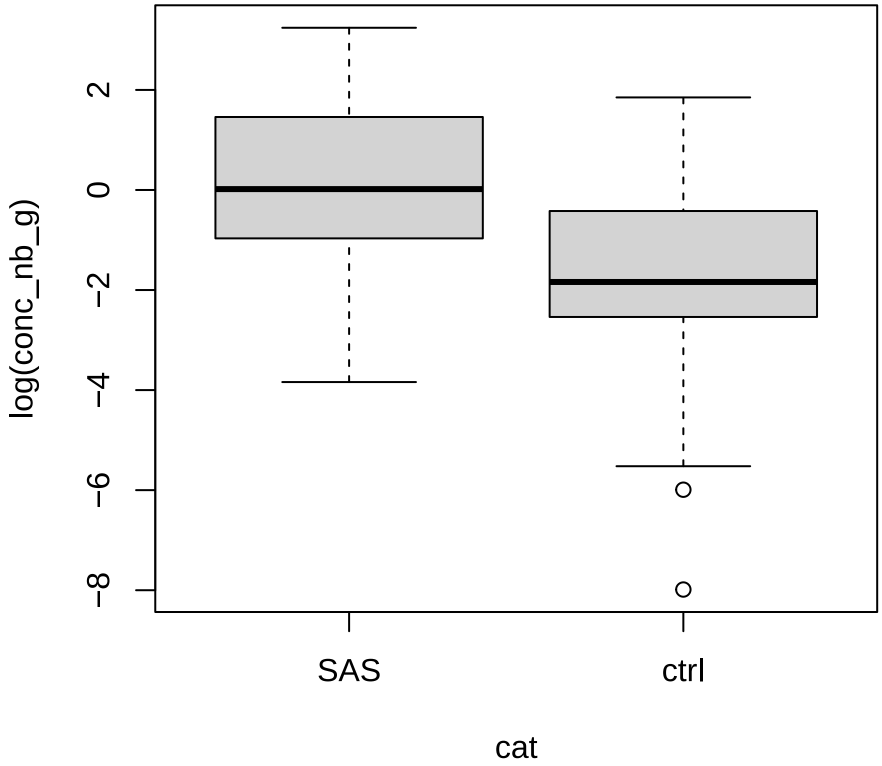


```r
# Wilcoxon test
wilcox.test(conc_nb_g ~ cat, alternative = "two.sided", exact = FALSE, data = a)
```

```
##
##  Wilcoxon rank sum test with continuity correction
##
## data:  conc_nb_g by cat
## W = 862, p-value = 0.0006681
## alternative hypothesis: true location shift is not equal to 0
```

```r
# T test
t.test(log(a$conc_nb_g) ~ a$cat, var.equal = TRUE)
```

```
##
##  Two Sample t-test
##
## data:  log(a$conc_nb_g) by a$cat
## t = 4.1004, df = 71, p-value = 0.0001083
## alternative hypothesis: true difference in means between group SAS and group ctrl is not equal to 0
## 95 percent confidence interval:
##  1.012386 2.928969
## sample estimates:
##  mean in group SAS mean in group ctrl
##          0.1053587         -1.8653188
```

```r
# -> SAS have significantly more MPs than ctrl fields
```

1.2 EED of SAS: model fits (Burr, log-normal, log-logistic, weibull, gamma)

```
###### SUBSET of SAS only (ie ctrls excluded):
a_sas <- subset(a, cat =="SAS") # we keep only lands spread with sludge (ie, we exclude ctrl fields)

# definition of the variable on which distributions will be fitted
conc_nb_g_sas <- a_sas$conc_nb_g

### Try to fit the data with different distributions
(fLN_eed_sas <- try(fitdist(conc_nb_g_sas, "lnorm"))) # log-normal fit
```

```
## Fitting of the distribution ' lnorm ' by maximum likelihood
## Parameters:
##          estimate Std. Error
## meanlog 0.1053587  0.2360316
## sdlog   1.6689952  0.1668992
```

```
(fLL_eed_sas <- try(fitdist(conc_nb_g_sas,"llogis"))) # log-logistic fit
```

```
## Fitting of the distribution ' llogis ' by maximum likelihood
## Parameters:
##       estimate Std. Error
## shape 1.033990  0.1210183
## scale 1.104251  0.2646502
```

```
(fB_eed_sas <- try(fitdist(conc_nb_g_sas, "burr"))) # Burr fit
```

```
## <simpleError in optim(par = vstart, fn = fnobj, fix.arg = fix.arg, obs = data,     gr = gradient, dd
## Error in fitdist(conc_nb_g_sas, "burr") :
##   the function mle failed to estimate the parameters,
##                 with the error code 100
```

```
## [1] "Error in fitdist(conc_nb_g_sas, \"burr\") : \n  the function mle failed to estimate the paramet
## attr(,"class")
## [1] "try-error"
## attr(,"condition")
## <simpleError in fitdist(conc_nb_g_sas, "burr"): the function mle failed to estimate the parameters,
##                 with the error code 100
## >
```

```
(fW_eed_sas <- try(fitdist(conc_nb_g_sas, "weibull"))) # Weibull fit
```

```
## Fitting of the distribution ' weibull ' by maximum likelihood
## Parameters:
##        estimate Std. Error
## shape 0.6424647 0.06833408
## scale 2.5456711 0.59386759
```

```
(fG_eed_sas <- try(fitdist(conc_nb_g_sas, "gamma"))) # gamma fit
```

```
## Fitting of the distribution ' gamma ' by maximum likelihood
## Parameters:
##        estimate Std. Error
## shape 0.5281624 0.08764516
## rate  0.1441949 0.03687455
```

```
# Comparison of fits, except Burr that fails
gofstat(list(fLN_eed_sas,
```

```
            fLL_eed_sas,
            fW_eed_sas,
            fG_eed_sas),
        fitnames = c("log-normal",
                     "log-logistic",
                     "Weibull",
                     "gamma"))
```

```
## Goodness-of-fit statistics
##                              log-normal log-logistic   Weibull     gamma
## Kolmogorov-Smirnov statistic 0.07576442   0.07551815 0.1181045 0.1626788
## Cramer-von Mises statistic   0.02961119   0.03144430 0.1342476 0.2796292
## Anderson-Darling statistic   0.23852489   0.25999143 0.8016592 1.4439764
##
## Goodness-of-fit criteria
##                                log-normal log-logistic  Weibull    gamma
## Akaike's Information Criterion   207.6519     209.5382 212.0618 215.9528
## Bayesian Information Criterion   211.4759     213.3623 215.8858 219.7768
```

```
# We select the distribution having the lowest AIC, which is: Log-normal
```

```
# Comparison of fits on cumulative distribution functions
# on a log scale of concentration for a better visualization
cdfcomp(list(fLN_eed_sas,
             fLL_eed_sas,
             fW_eed_sas,
             fG_eed_sas
             ), legendtext=c("log-normal",
                     "log-logistic",
                     "weibull",
                     "gamma"),
        xlab="nb/g dw in log scale", xlogscale = TRUE)
```

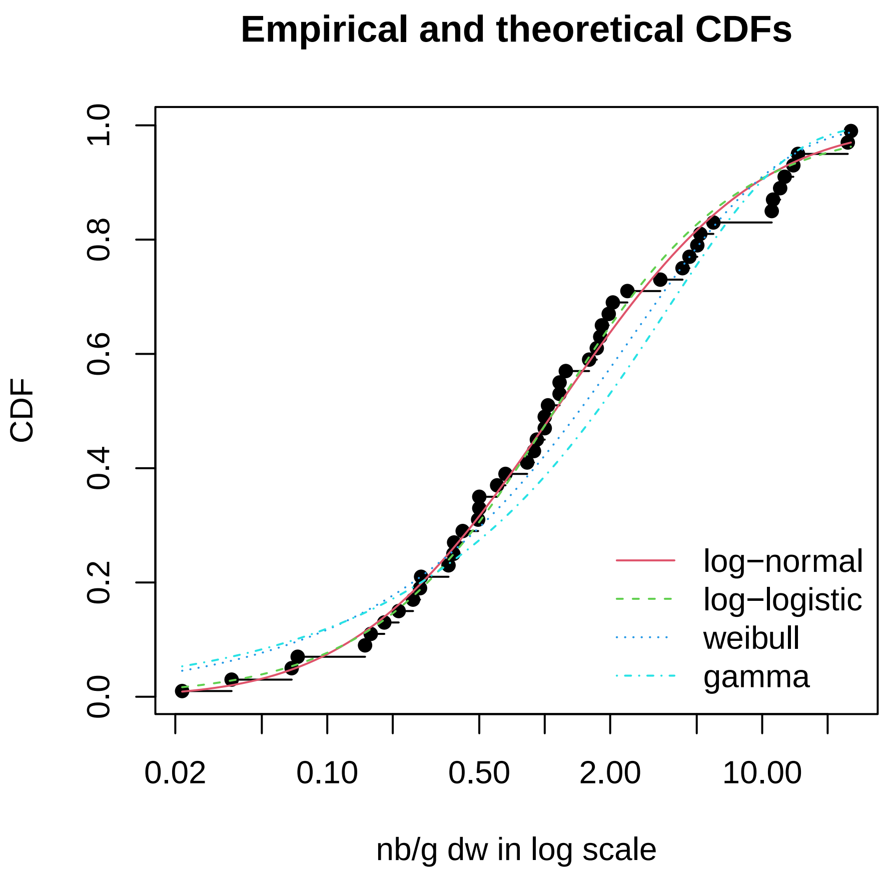


```
# Bootstrap of the best fit (lognormal distribution):
bLN_eed_sas <- bootdist(fLN_eed_sas, bootmethod = "param", niter=1001)
(EED_SAS_LN <- quantile(bLN_eed_sas, probs =  c(0.05, 0.5, 0.95)))
```

```
## (original) estimated quantiles for each specified probability (non-censored data)
##              p=0.05    p=0.5   p=0.95
## estimate 0.07136883 1.111109 17.29836
## Median of bootstrap estimates
##              p=0.05    p=0.5   p=0.95
## estimate 0.07428616 1.106164 16.63278
##
## two-sided 95 % CI of each quantile
##            p=0.05     p=0.5    p=0.95
## 2.5 %  0.03793309 0.7194334  8.368911
## 97.5 % 0.14689234 1.6865777 32.672405
```

```
## !! Caution, point estimate from the fit AND the median of bootstrap estimates are different !!

# For comparison, median on empirical data
median(conc_nb_g_sas)
```

```
## [1] 1.0175
```

1.3 EED of Control soils: model fits (Burr, log-normal, log-logistic, weibull, gamma)

```r
########## Selection of control soils
a_ctrls <- subset(a, cat =="ctrl")

# definition of the variable on which distributions will be fitted
conc_nb_g_ctrls <- a_ctrls$conc_nb_g

### Try to fit the data with different distributions
(fLN_eed_ctrls <- try(fitdist(conc_nb_g_ctrls, "lnorm"))) # log-normal fit
```

```
## Fitting of the distribution ' lnorm ' by maximum likelihood
## Parameters:
##          estimate Std. Error
## meanlog -1.865319  0.4744306
## sdlog    2.275289  0.3354728
```

```r
(fLL_eed_ctrls <- try(fitdist(conc_nb_g_ctrls,"llogis"))) # log-logistic fit
```

```
## Fitting of the distribution ' llogis ' by maximum likelihood
## Parameters:
##        estimate Std. Error
## shape 0.8113560 0.14376425
## scale 0.1835125 0.08083319
```

```r
(fB_eed_ctrls <- try(fitdist(conc_nb_g_ctrls, "burr"))) # Burr fit
```

```
## Fitting of the distribution ' burr ' by maximum likelihood
## Parameters:
##          estimate  Std. Error
## shape1 11.1310200  61.4028506
## shape2  0.5661534   0.1775626
## scale  27.6359891 318.9570397
```

```r
(fW_eed_ctrls <- try(fitdist(conc_nb_g_ctrls, "weibull"))) # Weibull fit
```

```
## Fitting of the distribution ' weibull ' by maximum likelihood
## Parameters:
##        estimate Std. Error
## shape 0.5380315 0.08652809
## scale 0.4426678 0.18094659
```

```r
(fG_eed_ctrls <- try(fitdist(conc_nb_g_ctrls, "gamma"))) # gamma fit
```

```
## Fitting of the distribution ' gamma ' by maximum likelihood
## Parameters:
##        estimate Std. Error
## shape 0.4027952 0.09614377
## rate  0.5086575 0.20656343
```

```r
gofstat(list(fLN_eed_ctrls,
             fLL_eed_ctrls,
             fB_eed_ctrls,
             fW_eed_ctrls,
             fG_eed_ctrls),
        fitnames = c("log-normal",
```

```r
                     "log-logistic",
                     "Burr",
                     "Weibull",
                     "gamma"))
```

```
## Goodness-of-fit statistics
##                               log-normal log-logistic       Burr    Weibull
## Kolmogorov-Smirnov statistic 0.18965384   0.13998136 0.13601740 0.13585067
## Cramer-von Mises statistic   0.09197372   0.05383916 0.06517005 0.06937932
## Anderson-Darling statistic   0.58027151   0.41922303 0.35624956 0.36955739
##                                  gamma
## Kolmogorov-Smirnov statistic 0.1506293
## Cramer-von Mises statistic   0.1155490
## Anderson-Darling statistic   0.5670855
##
## Goodness-of-fit criteria
##                                 log-normal log-logistic     Burr  Weibull
## Akaike's Information Criterion   21.28344     20.37047 20.67310 18.70460
## Bayesian Information Criterion   23.55443     22.64145 24.07959 20.97558
##                                   gamma
## Akaike's Information Criterion 20.13157
## Bayesian Information Criterion 22.40256
```

```r
# We select the distribution having the lowest AIC, which is: Weibull
```

```r
# Comparison of fits on cumulative distribution functions
# on a log scale of concentration for a better visualization
cdfcomp(list(fLN_eed_ctrls,
             fLL_eed_ctrls,
             fB_eed_ctrls,
             fW_eed_ctrls,
             fG_eed_ctrls
             ), legendtext=c("log-normal",
                     "log-logistic",
                     "burr",
                     "weibull",
                     "gamma"),
        xlab="nb/g dw in log scale", xlogscale = TRUE)
```

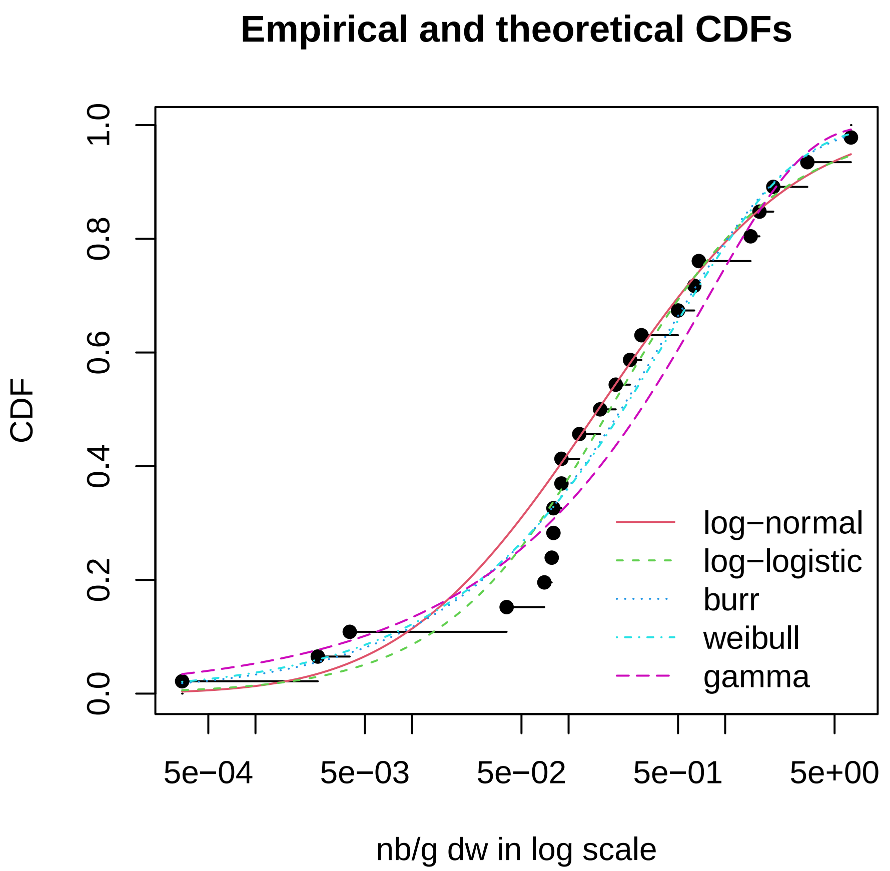


```
summary(fW_eed_ctrls)
```

```
## Fitting of the distribution ' weibull ' by maximum likelihood
## Parameters :
##        estimate Std. Error
## shape 0.5380315 0.08652809
## scale 0.4426678 0.18094659
## Loglikelihood:  -7.352298   AIC:  18.7046   BIC:  20.97558
## Correlation matrix:
##           shape     scale
## shape 1.0000000 0.3180221
## scale 0.3180221 1.0000000
```

```
# Bootstrap of the model:
bW_eed_ctrls <- bootdist(fW_eed_ctrls, bootmethod = "param", niter=1001)
(EED_ctrls_W<- quantile(bW_eed_ctrls, probs =  c(0.05, 0.5, 0.95)))
```

```
## (original) estimated quantiles for each specified probability (non-censored data)
##              p=0.05     p=0.5   p=0.95
## estimate 0.0017724 0.2239917 3.401883
## Median of bootstrap estimates
##                p=0.05     p=0.5   p=0.95
## estimate 0.002007002 0.2255439 3.138776
##
## two-sided 95 % CI of each quantile
##               p=0.05     p=0.5   p=0.95
## 2.5 %  0.0002243738 0.0862435 1.281381
```

```
## 97.5 % 0.0172985987 0.5436168 7.424880
## !! Caution, point estimate from the fit AND the median of bootstrap estimates are different !!

# For comparison, median on empirical data
median(conc_nb_g_ctrls)

## [1] 0.159
```

1.4 EED sludge (Burr, log-normal, log-logistic, weibull, gamma)

1.4.1 Undiluted concentrations (treated/stabilised sludge that could potentially be applied to lands)

```
b <- read.csv("sludge_conc_light.csv", stringsAsFactors = TRUE)
# file is already excluded from other units than mass/mass dw

sludge_conc_nb_g <- b$conc # here the units are already in nb/g dw

### Fit the data with different distributions:

(fLN_eed_sludge <- try(fitdist(sludge_conc_nb_g,"lnorm"))) # log-normal fit

## Fitting of the distribution ' lnorm ' by maximum likelihood
## Parameters:
##         estimate Std. Error
## meanlog 3.373347  0.2292942
## sdlog   1.972464  0.1621353

(fLL_eed_sludge <- try(fitdist(sludge_conc_nb_g,"llogis"))) # log-logistic fit

## Fitting of the distribution ' llogis ' by maximum likelihood
## Parameters:
##         estimate Std. Error
## shape  0.9706839 0.09679415
## scale 22.5443585 4.60462380

(fB_eed_sludge <- try(fitdist(sludge_conc_nb_g, "burr"))) # Burr fit

## Fitting of the distribution ' burr ' by maximum likelihood
## Parameters:
##         estimate Std. Error
## shape1 0.2565312 0.08672958
## shape2 2.1460068 0.51548356
## scale  5.5920882 1.55501106

(fW_eed_sludge <- try(fitdist(sludge_conc_nb_g, "weibull"))) # Weibull fit

## Fitting of the distribution ' weibull ' by maximum likelihood
## Parameters:
##         estimate  Std. Error
## shape  0.4134736  0.03248017
## scale 86.8682405 26.04560712

(fG_eed_sludge <- try(fitdist(sludge_conc_nb_g, "gamma"))) # gamma fit

## <simpleError in optim(par = vstart, fn = fnobj, fix.arg = fix.arg, obs = data,     gr = gradient, dd
## Error in fitdist(sludge_conc_nb_g, "gamma") :
```

```
##   the function mle failed to estimate the parameters,
##                 with the error code 100

## [1] "Error in fitdist(sludge_conc_nb_g, \"gamma\") : \n  the function mle failed to estimate the para
## attr(,"class")
## [1] "try-error"
## attr(,"condition")
## <simpleError in fitdist(sludge_conc_nb_g, "gamma"): the function mle failed to estimate the paramete
##                 with the error code 100
## >
```

```
gofstat(list(fLN_eed_sludge,
             fLL_eed_sludge,
             fB_eed_sludge,
             fW_eed_sludge),
        fitnames = c("log-normal",
                     "log-logistic",
                     "burr",
                     "weibull"))
```

```
## Goodness-of-fit statistics
##                              log-normal log-logistic       burr   weibull
## Kolmogorov-Smirnov statistic  0.1393037   0.07750463 0.04968112 0.2032508
## Cramer-von Mises statistic    0.4053056   0.12653764 0.02871778 1.0040992
## Anderson-Darling statistic    2.5727805   1.45567575 0.26733270 5.7488721
##
## Goodness-of-fit criteria
##                                log-normal log-logistic     burr  weibull
## Akaike's Information Criterion   813.7922     807.6019 793.8575 848.7313
## Bayesian Information Criterion   818.4003     812.2100 800.7697 853.3394
```

```
# We select the distribution having the lowest AIC, which is: Burr
```

```
# Comparison of fits on cumulative distribution functions
# on a log scale of concentration for a better visualization
cdfcomp(list(fLN_eed_sludge,
             fLL_eed_sludge,
             fB_eed_sludge,
             fW_eed_sludge
             ), legendtext=c("log-normal",
                     "log-logistic",
                     "burr",
                     "weibull"),
        xlab="nb/g dw in log scale", xlogscale=TRUE)
```

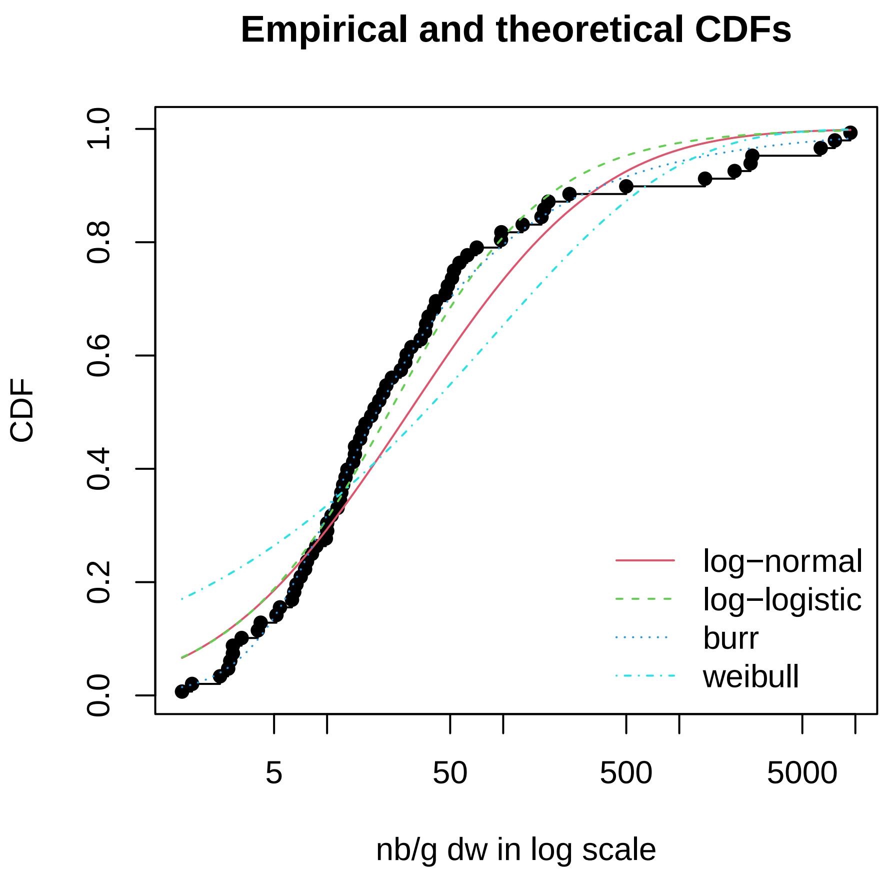


```
# Estimates from the bootstrap:
bB_eed_sludge <- bootdist(fB_eed_sludge, bootmethod = "param", niter=1001)
(EED_sludge_B <- quantile(bB_eed_sludge, probs =  c(0.01,0.02, 0.05, 0.5, 0.95)))
```

```
## (original) estimated quantiles for each specified probability (non-censored data)
##            p=0.01   p=0.02   p=0.05    p=0.5   p=0.95
## estimate 1.247193 1.742917 2.769395 19.06938 1290.795
## Median of bootstrap estimates
##            p=0.01   p=0.02   p=0.05    p=0.5   p=0.95
## estimate 1.347374 1.848911 2.864978 18.92326 1235.519
##
## two-sided 95 % CI of each quantile
##           p=0.01    p=0.02   p=0.05    p=0.5    p=0.95
## 2.5 %  0.5810673 0.9427866 1.786058 13.32051  324.3722
## 97.5 % 2.6921451 3.2517478 4.277383 28.51882 5573.3975
##
## The estimation method converged only for 1000 among 1001 bootstrap iterations.
```

```
# For comparison, median on empirical data
median(sludge_conc_nb_g)
```

```
## [1] 18.205
```

2 SSD on soil species (log-normal, log-logistic and Burr)

2.1 Averaged by species

```
d <- read.csv("SSD_dataset_simplified_r.csv", header = TRUE, sep = ",", stringsAsFactors = TRUE)

# b$NOEC_eq_nb_kg # values already normalized in the spreadsheet as nb_kg divided by overall UFs
# NOEC_eq correspond to doses that do not induce effect on a chronic exposure basis
# for the least severe endpoint category, accounting for uncertainties factor harmonization.
# So it is a conservative toxicological value.

d$NOEC_eq_nb_g <- d$NOEC_eq_nb_kg/1000 # to not have high numerical values for better handling in R, it

# calculate the geometric mean by species:
res <- d %>%
  group_by(Entity) %>%
  summarize(X = exp(mean(log(NOEC_eq_nb_g)))) # note: log here is neperian logarithm
# the resulting NOEC_eq geometric means in nb/g are in res$X
# write.csv(res, "res.csv")


#### Fit of distributions:

(fLN_ssd_avg <- try(fitdist(res$X,"lnorm"))) # log-normal fit
```

```
## Fitting of the distribution ' lnorm ' by maximum likelihood
## Parameters:
##         estimate Std. Error
## meanlog 4.410179  0.7968093
## sdlog   3.984047  0.5634291
```

```
(fLL_ssd_avg <- try(fitdist(res$X,"llogis"))) # log-logistic fit
```

```
## Fitting of the distribution ' llogis ' by maximum likelihood
## Parameters:
##         estimate   Std. Error
## shape  0.4160008  0.06702035
## scale 74.6285148 64.13357246
```

```
(fB_ssd_avg <- try(fitdist(res$X,"burr"))) # Burr fit
```

```
## Fitting of the distribution ' burr ' by maximum likelihood
## Parameters:
##         estimate Std. Error
## shape1 0.3512269  0.5831497
## shape2 0.7058155  0.7776937
## scale  2.7368029 10.2727670
```

```
gofstat(list(fLN_ssd_avg,
             fLL_ssd_avg,
             fB_ssd_avg),
        fitnames = c("log-normal",
                     "log-logistic",
                     "burr"))
```

```
## Goodness-of-fit statistics
```

```
##                               log-normal log-logistic      burr
## Kolmogorov-Smirnov statistic 0.14071060   0.13675397 0.1453625
## Cramer-von Mises statistic   0.08870931   0.09388715 0.1012175
## Anderson-Darling statistic   0.47910864   0.52602433 0.5593725
##
## Goodness-of-fit criteria
##                                 log-normal log-logistic     burr
## Akaike's Information Criterion   364.5708     366.5674 368.5287
## Bayesian Information Criterion   367.0085     369.0051 372.1853
```

```
# Based on the AIC: the log-normal fit is the best. So it is selected.

# Comparison of fits on cumulative distribution functions
# on a log scale of concentration for a better visualization
cdfcomp(list(fLN_ssd_avg,
             fLL_ssd_avg,
             fB_ssd_avg),
        legendtext=c("log-normal", "log-logistic", "burr"),
        xlab="nb/g dw in log scale",
        xlogscale = TRUE)
```

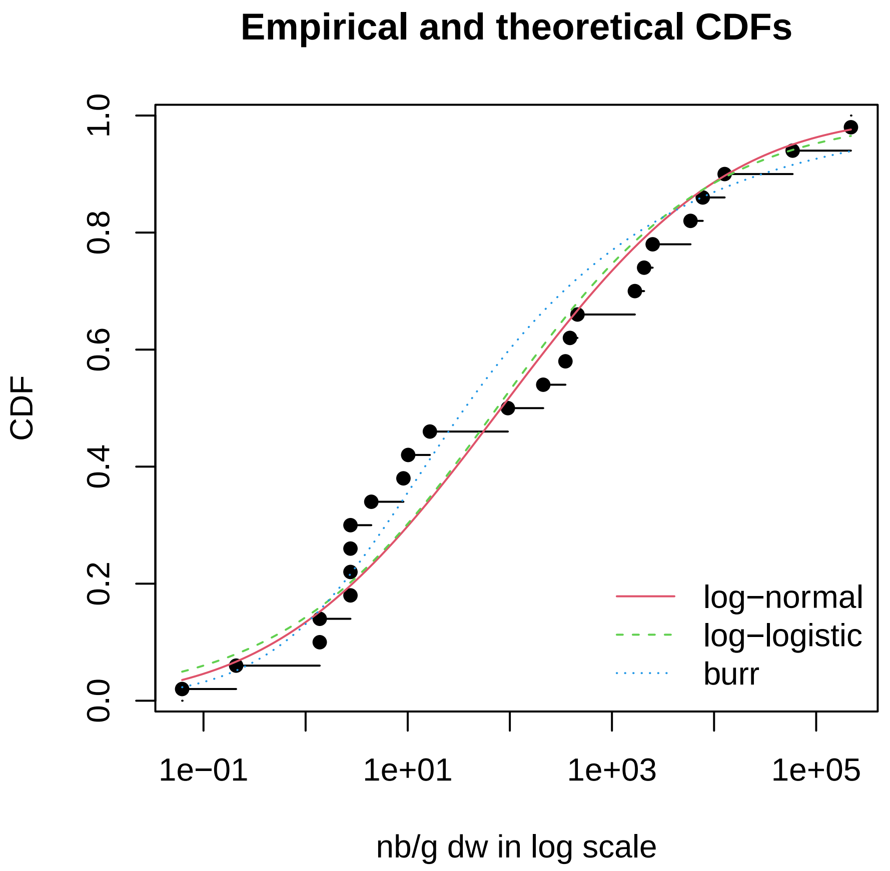


```
# Estimates of the bootstrap:
bLN_ssd_avg <- bootdist(fLN_ssd_avg, bootmethod = "param", niter=1001)
(estimates<- quantile(bLN_ssd_avg,
                      probs = c(0.05, 0.1, 0.2, 0.3, 0.4, 0.5, 0.95)))
```

```
## (original) estimated quantiles for each specified probability (non-censored data)
##             p=0.05     p=0.1     p=0.2     p=0.3     p=0.4     p=0.5    p=0.95
```

```
## estimate 0.117303 0.4987839 2.878072 10.18527 29.98905 82.28418 57719.62
## Median of bootstrap estimates
##              p=0.05     p=0.1    p=0.2   p=0.3    p=0.4    p=0.5   p=0.95
## estimate 0.1455021 0.5979728 3.339944 11.2441 32.05159 85.48437 47202.98
##
## two-sided 95 % CI of each quantile
##             p=0.05      p=0.1      p=0.2     p=0.3      p=0.4     p=0.5
## 2.5 %   0.01330982 0.07181517  0.5351993  2.288197   7.046892  19.45781
## 97.5 % 1.41574332 4.54102635 18.4520215 59.119441 155.454482 389.01762
##             p=0.95
## 2.5 %     4336.356
## 97.5 % 509802.430
```

3 Monte Carlo simulations for different sludge dilution factors

3.1 Uncertainty dimension

```r
# specification of uncertainty and variability dimensions
ndunc(1001)
```

```
## [1] 1001
```

```r
ndvar(1001)
```

```
## [1] 1001
```

```r
# Retrieval of the bootstrap results for sludge
# (from the fit of the Burr distribution)
boot_sludge_conc <- bB_eed_sludge

# uncertainty on the exposition
expo_conc_shape1 <- mcdata(boot_sludge_conc$estim$shape1, type = "U")
expo_conc_shape2 <- mcdata(boot_sludge_conc$estim$shape2, type = "U")
expo_conc_scale <- mcdata(boot_sludge_conc$estim$scale, type = "U")

# Retrieval of the bootstrap results for SSD
# (from the fit of the lognormal distribution)
boot_NOEC_eq_nb_g <- bLN_ssd_avg

# uncertainty on the tox value
NOEC_eq_nb_g_meanlog <- mcdata(boot_NOEC_eq_nb_g$estim$meanlog, type = "U")
NOEC_eq_nb_g_sdlog <- mcdata(boot_NOEC_eq_nb_g$estim$sdlog, type = "U")
```

3.2 Code to simulate variability conditionally to uncertainty (on 1 scenario)

```r
# variability on the exposition
expo_conc <- mcstoc(rburr, type = "VU", shape1 = expo_conc_shape1,
                    shape2 = expo_conc_shape2, scale = expo_conc_scale)

summary(expo_conc)
```

```
## node :
##             mean       sd    Min 2.5%   25%  50%   75% 97.5%      Max  nsv Na's
## median 5.51e+03 1.07e+05 0.3979 2.09  7.87 18.9  68.9  4096 3.12e+06 1001    0
## mean   1.79e+08 5.65e+09 0.5062 2.14  8.01 19.6  73.6  7244 1.79e+11 1001    1
```

```
## 2.5%    1.67e+02 1.21e+03 0.0385 1.04  5.49 13.0  40.0   699 2.70e+04 1001    0
## 97.5%  7.00e+06 2.21e+08 1.5990 3.56 11.08 29.4 137.5 33801 6.98e+09 1001    0
```

```
plot(log10(expo_conc))
```

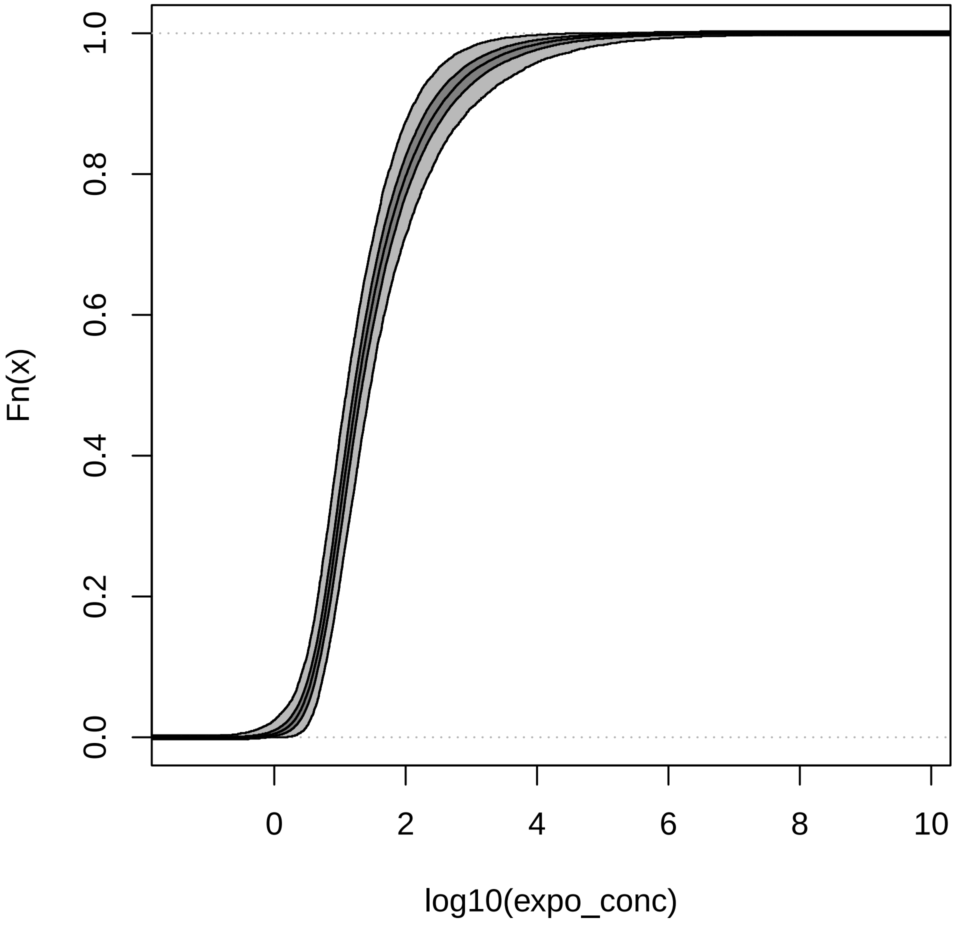


```
# variability on the tox value
tox_value <- mcstoc(rlnorm, type = "VU", meanlog = NOEC_eq_nb_g_meanlog,
                    sdlog = NOEC_eq_nb_g_sdlog)
summary(tox_value)
```

```
## node :
##             mean       sd      Min    2.5%   25%   50%  75%   97.5%      Max  nsv
## median   63076 8.55e+05 3.93e-04 0.04561  6.51  85.5 1169  154533 2.36e+07 1001
## mean   1456894 4.00e+07 2.55e-03 0.10742  9.45 119.9 1743  455756 1.24e+09 1001
## 2.5%      1984 1.84e+04 1.98e-06 0.00292  1.10  18.2  200   11548 3.38e+05 1001
## 97.5%  5682414 1.48e+08 1.87e-02 0.58943 35.55 420.9 7001 2729535 4.29e+09 1001
##        Na's
## median    0
## mean      0
## 2.5%      0
## 97.5%     0
```

```
plot(log10(tox_value))
```

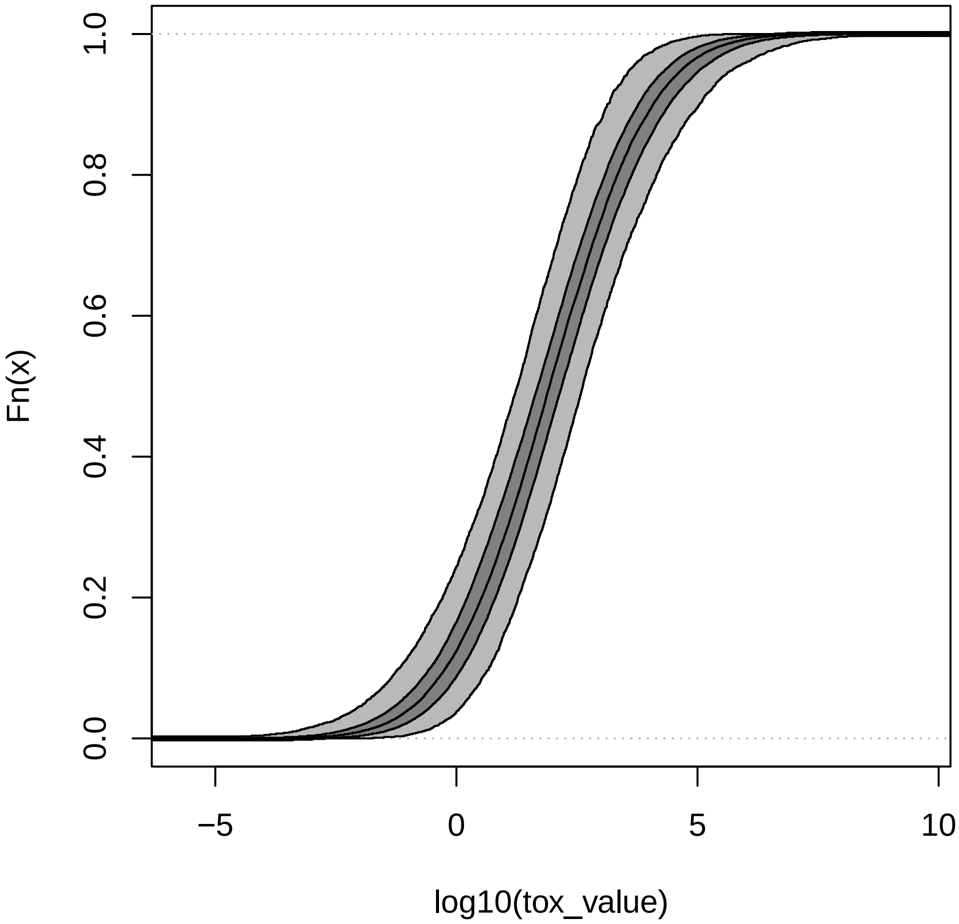


```r
# species in the SSD affected by the exposition
affected_dil1 <- expo_conc > tox_value
summary(affected_dil1)
```

```
## node :
##          mean    sd Min 2.5% 25%   50%   75% 97.5% Max  nsv Na's
## median 0.393 0.488   0    0   0 0.000 1.000     1   1 1001    0
## mean   0.394 0.482   0    0   0 0.076 0.966     1   1 1001    1
## 2.5%   0.243 0.429   0    0   0 0.000 0.000     1   1 1001    0
## 97.5%  0.545 0.500   0    0   0 1.000 1.000     1   1 1001    0
```

3.3 Repeats for 17 dilution scenarios

```r
affected_dil2 <- expo_conc / 2 > tox_value
affected_dil3 <- expo_conc / 3 > tox_value
affected_dil4 <- expo_conc / 4 > tox_value
affected_dil5 <- expo_conc / 5 > tox_value
affected_dil6 <- expo_conc / 6 > tox_value
affected_dil7 <- expo_conc / 7 > tox_value
affected_dil8 <- expo_conc / 8 > tox_value
affected_dil9 <- expo_conc / 9 > tox_value
affected_dil10 <- expo_conc / 10 > tox_value
affected_dil11 <- expo_conc / 11 > tox_value
affected_dil12 <- expo_conc / 12 > tox_value
```

```r
affected_dil13 <- expo_conc / 13 > tox_value
affected_dil14 <- expo_conc / 14 > tox_value
affected_dil15 <- expo_conc / 15 > tox_value
affected_dil16 <- expo_conc / 16 > tox_value
affected_dil17 <- expo_conc / 17 > tox_value
```

```r
# gathering the nodes of the model
# rows correspond to the variability with the mean, sd, min,...
# columns correspond to uncertainty with 2.5 and 97.5% quantiles

model <- mc(expo_conc, tox_value,
            affected_dil1,
            affected_dil2,
            affected_dil3,
            affected_dil4,
            affected_dil5,
            affected_dil6,
            affected_dil7,
            affected_dil8,
            affected_dil9,
            affected_dil10,
            affected_dil11,
            affected_dil12,
            affected_dil13,
            affected_dil14,
            affected_dil15,
            affected_dil16,
            affected_dil17)

(summary_res <- summary(model))
```

```
## expo_conc :
##             mean       sd    Min 2.5%   25%  50%   75% 97.5%      Max  nsv Na's
## median 5.51e+03 1.07e+05 0.3979 2.09  7.87 18.9  68.9  4096 3.12e+06 1001    0
## mean   1.79e+08 5.65e+09 0.5062 2.14  8.01 19.6  73.6  7244 1.79e+11 1001    1
## 2.5%   1.67e+02 1.21e+03 0.0385 1.04  5.49 13.0  40.0   699 2.70e+04 1001    0
## 97.5%  7.00e+06 2.21e+08 1.5990 3.56 11.08 29.4 137.5 33801 6.98e+09 1001    0
##
## tox_value :
##           mean       sd      Min    2.5%   25%   50%  75%   97.5%      Max  nsv
## median   63076 8.55e+05 3.93e-04 0.04561  6.51  85.5 1169  154533 2.36e+07 1001
## mean   1456894 4.00e+07 2.55e-03 0.10742  9.45 119.9 1743  455756 1.24e+09 1001
## 2.5%      1984 1.84e+04 1.98e-06 0.00292  1.10  18.2  200   11548 3.38e+05 1001
## 97.5%  5682414 1.48e+08 1.87e-02 0.58943 35.55 420.9 7001 2729535 4.29e+09 1001
##        Na's
## median    0
## mean      0
## 2.5%      0
## 97.5%     0
##
## affected_dil1 :
##         mean    sd Min 2.5% 25%   50%   75% 97.5% Max  nsv Na's
## median 0.393 0.488   0    0   0 0.000 1.000     1   1 1001    0
## mean   0.394 0.482   0    0   0 0.076 0.966     1   1 1001    1
```

```
## 2.5%   0.243 0.429   0    0   0 0.000 0.000     1   1 1001    0
## 97.5%  0.545 0.500   0    0   0 1.000 1.000     1   1 1001    0
##
## affected_dil2 :
##         mean    sd Min 2.5% 25%   50%   75% 97.5% Max  nsv Na's
## median 0.334 0.472   0    0   0 0.000 1.000     1   1 1001    0
## mean   0.335 0.466   0    0   0 0.017 0.873     1   1 1001    1
## 2.5%   0.192 0.394   0    0   0 0.000 0.000     1   1 1001    0
## 97.5%  0.489 0.500   0    0   0 0.000 1.000     1   1 1001    0
##
## affected_dil3 :
##         mean    sd Min 2.5% 25%   50%   75% 97.5% Max  nsv Na's
## median 0.300 0.458   0    0   0 0.000 1.000     1   1 1001    0
## mean   0.302 0.453   0    0   0 0.006 0.768     1   1 1001    1
## 2.5%   0.164 0.370   0    0   0 0.000 0.000     1   1 1001    0
## 97.5%  0.447 0.497   0    0   0 0.000 1.000     1   1 1001    0
##
## affected_dil4 :
##         mean    sd Min 2.5% 25%   50%   75% 97.5% Max  nsv Na's
## median 0.277 0.448   0    0   0 0.000 1.000     1   1 1001    0
## mean   0.280 0.443   0    0   0 0.002 0.654     1   1 1001    1
## 2.5%   0.148 0.355   0    0   0 0.000 0.000     1   1 1001    0
## 97.5%  0.424 0.494   0    0   0 0.000 1.000     1   1 1001    0
##
## affected_dil5 :
##         mean    sd Min 2.5% 25%   50%   75% 97.5% Max  nsv Na's
## median 0.260 0.439   0    0   0 0.000 1.000     1   1 1001    0
## mean   0.264 0.434   0    0   0 0.001 0.561     1   1 1001    1
## 2.5%   0.136 0.343   0    0   0 0.000 0.000     1   1 1001    0
## 97.5%  0.405 0.491   0    0   0 0.000 1.000     1   1 1001    0
##
## affected_dil6 :
##         mean    sd Min 2.5% 25%   50%   75% 97.5% Max  nsv Na's
## median 0.247 0.431   0    0   0 0.000 0.000     1   1 1001    0
## mean   0.251 0.427   0    0   0 0.001 0.481     1   1 1001    1
## 2.5%   0.125 0.331   0    0   0 0.000 0.000     1   1 1001    0
## 97.5%  0.387 0.487   0    0   0 0.000 1.000     1   1 1001    0
##
## affected_dil7 :
##         mean    sd Min 2.5% 25% 50%   75% 97.5% Max  nsv Na's
## median 0.236 0.425   0    0   0   0 0.000     1   1 1001    0
## mean   0.240 0.420   0    0   0   0 0.427     1   1 1001    1
## 2.5%   0.117 0.321   0    0   0   0 0.000     1   1 1001    0
## 97.5%  0.372 0.483   0    0   0   0 1.000     1   1 1001    0
##
## affected_dil8 :
##         mean    sd Min 2.5% 25% 50%   75% 97.5% Max  nsv Na's
## median 0.227 0.419   0    0   0   0 0.000     1   1 1001    0
## mean   0.231 0.414   0    0   0   0 0.374     1   1 1001    1
## 2.5%   0.112 0.315   0    0   0   0 0.000     1   1 1001    0
## 97.5%  0.363 0.481   0    0   0   0 1.000     1   1 1001    0
##
## affected_dil9 :
##         mean    sd Min 2.5% 25% 50%   75% 97.5% Max  nsv Na's
```

```
## median 0.219 0.414   0    0   0   0 0.000     1   1 1001    0
## mean   0.223 0.409   0    0   0   0 0.333     1   1 1001    1
## 2.5%   0.108 0.310   0    0   0   0 0.000     1   1 1001    0
## 97.5%  0.354 0.478   0    0   0   0 1.000     1   1 1001    0
##
## affected_dil10 :
##         mean    sd Min 2.5% 25% 50%   75% 97.5% Max  nsv Na's
## median 0.212 0.409   0    0   0   0 0.000     1   1 1001    0
## mean   0.216 0.405   0    0   0   0 0.289     1   1 1001    1
## 2.5%   0.102 0.303   0    0   0   0 0.000     1   1 1001    0
## 97.5%  0.345 0.475   0    0   0   0 1.000     1   1 1001    0
##
## affected_dil11 :
##          mean    sd Min 2.5% 25% 50%   75% 97.5% Max  nsv Na's
## median 0.2053 0.404   0    0   0   0 0.000     1   1 1001    0
## mean   0.2100 0.400   0    0   0   0 0.248     1   1 1001    1
## 2.5%   0.0989 0.299   0    0   0   0 0.000     1   1 1001    0
## 97.5%  0.3367 0.473   0    0   0   0 1.000     1   1 1001    0
##
## affected_dil12 :
##          mean    sd Min 2.5% 25% 50%   75% 97.5% Max  nsv Na's
## median 0.2008 0.401   0    0   0   0 0.000     1   1 1001    0
## mean   0.2046 0.396   0    0   0   0 0.222     1   1 1001    1
## 2.5%   0.0929 0.290   0    0   0   0 0.000     1   1 1001    0
## 97.5%  0.3297 0.470   0    0   0   0 1.000     1   1 1001    0
##
## affected_dil13 :
##          mean    sd Min 2.5% 25% 50%   75% 97.5% Max  nsv Na's
## median 0.1958 0.397   0    0   0   0 0.000     1   1 1001    0
## mean   0.1996 0.393   0    0   0   0 0.201     1   1 1001    1
## 2.5%   0.0899 0.286   0    0   0   0 0.000     1   1 1001    0
## 97.5%  0.3237 0.468   0    0   0   0 1.000     1   1 1001    0
##
## affected_dil14 :
##          mean    sd Min 2.5% 25% 50%   75% 97.5% Max  nsv Na's
## median 0.1903 0.393   0    0   0   0 0.000     1   1 1001    0
## mean   0.1951 0.389   0    0   0   0 0.179     1   1 1001    1
## 2.5%   0.0879 0.283   0    0   0   0 0.000     1   1 1001    0
## 97.5%  0.3197 0.467   0    0   0   0 1.000     1   1 1001    0
##
## affected_dil15 :
##          mean    sd Min 2.5% 25% 50%   75% 97.5% Max  nsv Na's
## median 0.1868 0.390   0    0   0   0 0.000     1   1 1001    0
## mean   0.1909 0.386   0    0   0   0 0.157     1   1 1001    1
## 2.5%   0.0859 0.280   0    0   0   0 0.000     1   1 1001    0
## 97.5%  0.3147 0.465   0    0   0   0 1.000     1   1 1001    0
##
## affected_dil16 :
##          mean    sd Min 2.5% 25% 50%   75% 97.5% Max  nsv Na's
## median 0.1838 0.388   0    0   0   0 0.000     1   1 1001    0
## mean   0.1872 0.383   0    0   0   0 0.143     1   1 1001    1
## 2.5%   0.0849 0.279   0    0   0   0 0.000     1   1 1001    0
## 97.5%  0.3097 0.463   0    0   0   0 1.000     1   1 1001    0
##
```

```
## affected_dil17 :
##           mean    sd Min 2.5% 25% 50%   75% 97.5% Max  nsv Na's
## median 0.1798 0.384   0    0   0   0 0.000     1   1 1001    0
## mean   0.1836 0.380   0    0   0   0 0.129     1   1 1001    1
## 2.5%   0.0819 0.274   0    0   0   0 0.000     1   1 1001    0
## 97.5%  0.3047 0.461   0    0   0   0 1.000     1   1 1001    0
```

```
# for each scenario, for each node affected_dil..,
# the column mean corresponds to the estimation
# (point estimation and 95% interval estimation)
# of the proportion of affected species
# as in the model affected is equal to 1 if the species is affected
# and to 0 if it is not affected
```

4 Monte Carlo simulations for control soils

```
# 1 chunk:

# Retrieval of the bootstrap results for control soils
# (from the fit of the Weibull distribution)
boot_ctrls_conc <- bW_eed_ctrls

## MC2D: Uncertainty dimension
# specification of uncertainty and variability dimensions
ndunc(1001)
```

```
## [1] 1001
```

```
ndvar(1001)
```

```
## [1] 1001
```

```
# uncertainty on the exposition
expo_conc_shape <- mcdata(boot_ctrls_conc$estim$shape, type = "U")
expo_conc_scale <- mcdata(boot_ctrls_conc$estim$scale, type = "U")

## Code to simulate variability conditionally to uncertainty
# variability on the exposition
expo_conc <- mcstoc(rweibull, type = "VU", shape = expo_conc_shape, scale = expo_conc_scale)

#summary(expo_conc)
plot(log10(expo_conc))
```

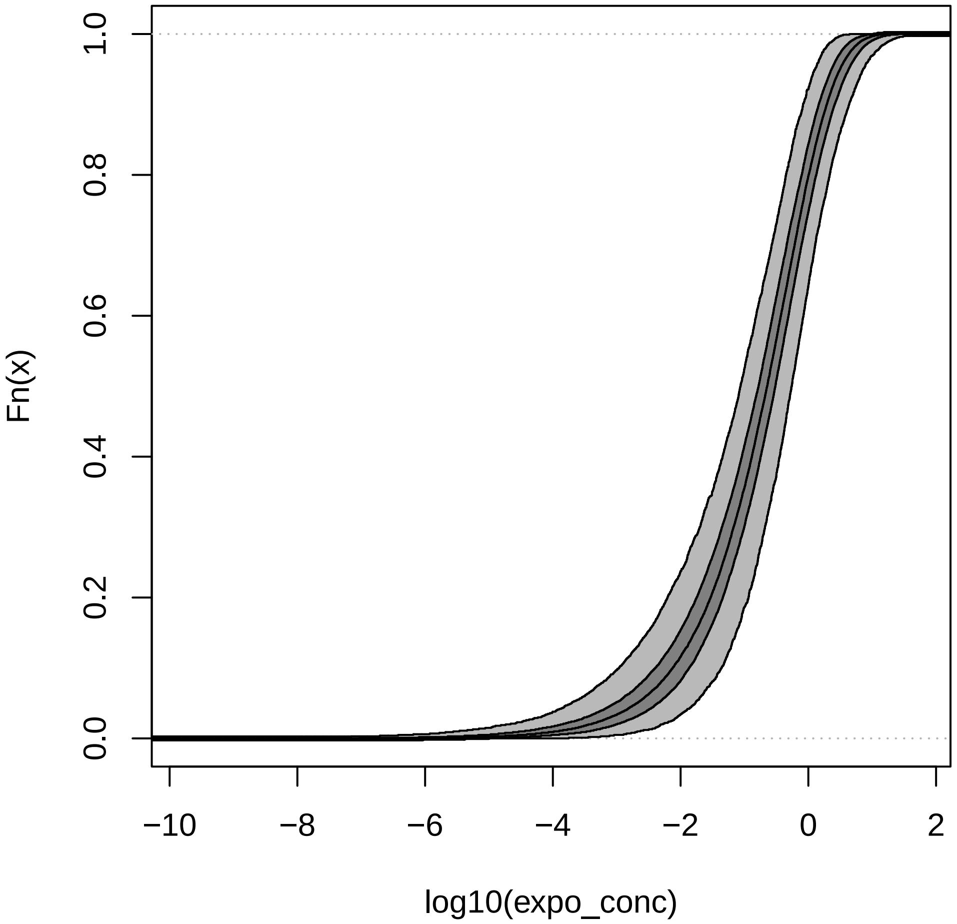


```
# species in the SSD affected by the exposition
affected <- expo_conc > tox_value
model <- mc(expo_conc, tox_value, affected)
(summary_res_ctrl <- summary(model))
```

```
## expo_conc :
##         mean    sd      Min     2.5%    25%    50%   75% 97.5%  Max  nsv Na's
## median 0.740 1.408 9.03e-07 6.01e-04 0.0463 0.2269 0.777  4.52 15.6 1001    0
## mean   0.803 1.625 2.15e-05 1.33e-03 0.0554 0.2465 0.836  5.02 20.3 1001    0
## 2.5%   0.318 0.492 1.53e-10 3.81e-05 0.0121 0.0871 0.347  1.71  4.4 1001    0
## 97.5%  1.673 3.745 1.77e-04 7.50e-03 0.1606 0.5497 1.627 11.17 57.9 1001    0
##
## tox_value :
##            mean       sd      Min    2.5%   25%   50%  75%   97.5%      Max  nsv
## median    63076 8.55e+05 3.93e-04 0.04561  6.51  85.5 1169  154533 2.36e+07 1001
## mean    1456894 4.00e+07 2.55e-03 0.10742  9.45 119.9 1743  455756 1.24e+09 1001
## 2.5%       1984 1.84e+04 1.98e-06 0.00292  1.10  18.2  200   11548 3.38e+05 1001
## 97.5%   5682414 1.48e+08 1.87e-02 0.58943 35.55 420.9 7001 2729535 4.29e+09 1001
##        Na's
## median    0
## mean      0
## 2.5%      0
## 97.5%     0
##
## affected :
```

```
##          mean    sd Min 2.5% 25% 50% 75% 97.5% Max  nsv Na's
## median 0.0779 0.268   0    0   0   0   0 1.000   1 1001    0
## mean   0.0811 0.263   0    0   0   0   0 0.953   1 1001    0
## 2.5%   0.0200 0.140   0    0   0   0   0 0.000   1 1001    0
## 97.5%  0.1708 0.377   0    0   0   0   0 1.000   1 1001    0
```

5 Monte Carlo simulations for SAS

```
# 1 chunk:
## Uncertainty dimension

# specification of uncertainty and variability dimensions
ndunc(1001)
```

```
## [1] 1001
```

```
ndvar(1001)
```

```
## [1] 1001
```

```
# Retrieval of the bootstrap results for sludge
# (from the fit of the LN distribution)
boot_SAS_conc <- bLN_eed_sas

# uncertainty on the exposition
boot_SAS_conc_meanlog <- mcdata(boot_SAS_conc$estim$meanlog, type = "U")
boot_SAS_conc_sdlog <- mcdata(boot_SAS_conc$estim$sdlog, type = "U")


## Code to simulate variability conditionally to uncertainty

# variability on the exposition
expo_conc <- mcstoc(rlnorm, type = "VU", meanlog = boot_SAS_conc_meanlog,
                    sdlog = boot_SAS_conc_sdlog)

summary(expo_conc)
```

```
## node :
##        mean   sd      Min   2.5%   25%   50%  75% 97.5%  Max  nsv Na's
## median 4.26 12.4 0.005713 0.0444 0.364 1.105 3.33  27.3  212 1001    0
## mean   4.63 16.5 0.007226 0.0483 0.378 1.138 3.47  30.2  341 1001    0
## 2.5%   2.22  4.6 0.000935 0.0188 0.220 0.683 2.05  12.0   58 1001    0
## 97.5%  9.23 50.0 0.021378 0.0978 0.613 1.713 5.41  62.2 1383 1001    0
```

```
plot(log10(expo_conc))
```

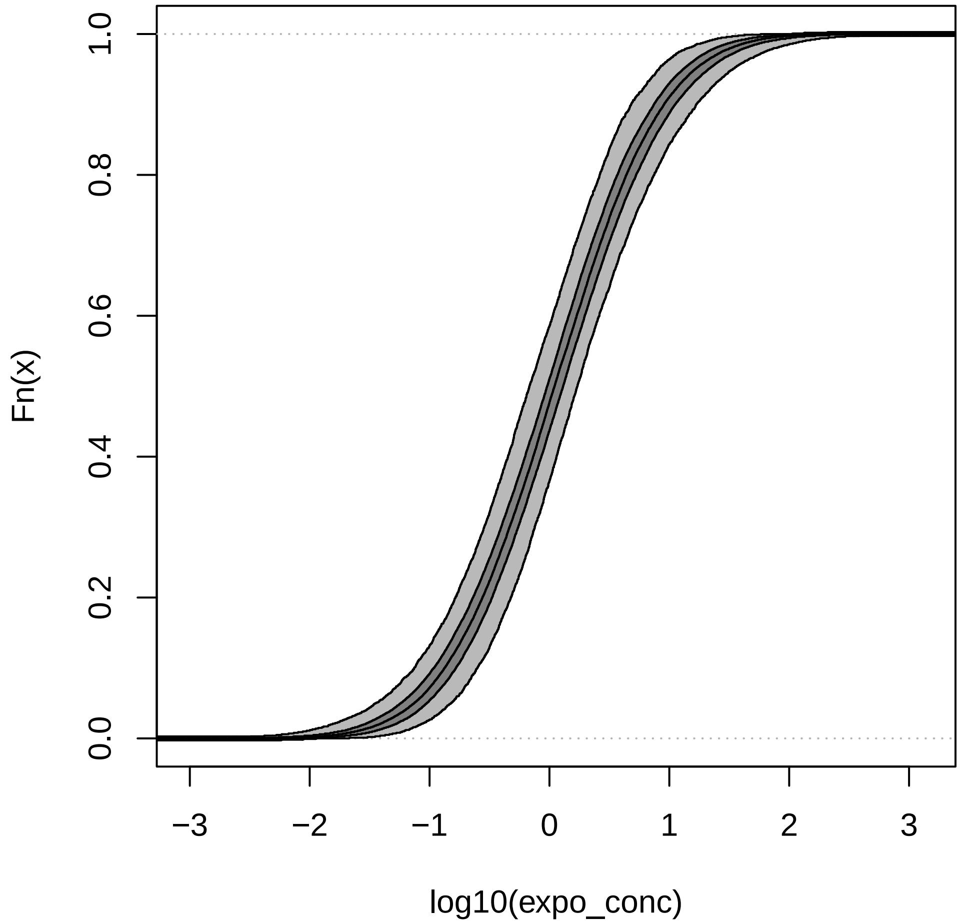


```r
# species in the SSD affected by the exposition
affected <- expo_conc > tox_value
model <- mc(expo_conc, tox_value, affected)
(summary_res_SAS <- summary(model))
```

```
## expo_conc :
##        mean   sd      Min   2.5%   25%   50%  75% 97.5%  Max  nsv Na's
## median 4.26 12.4 0.005713 0.0444 0.364 1.105 3.33  27.3  212 1001    0
## mean   4.63 16.5 0.007226 0.0483 0.378 1.138 3.47  30.2  341 1001    0
## 2.5%   2.22  4.6 0.000935 0.0188 0.220 0.683 2.05  12.0   58 1001    0
## 97.5%  9.23 50.0 0.021378 0.0978 0.613 1.713 5.41  62.2 1383 1001    0
##
## tox_value :
##           mean       sd      Min    2.5%   25%   50%  75%   97.5%      Max  nsv
## median   63076 8.55e+05 3.93e-04 0.04561  6.51  85.5 1169  154533 2.36e+07 1001
## mean   1456894 4.00e+07 2.55e-03 0.10742  9.45 119.9 1743  455756 1.24e+09 1001
## 2.5%      1984 1.84e+04 1.98e-06 0.00292  1.10  18.2  200   11548 3.38e+05 1001
## 97.5%  5682414 1.48e+08 1.87e-02 0.58943 35.55 420.9 7001 2729535 4.29e+09 1001
##        Na's
## median    0
## mean      0
## 2.5%      0
## 97.5%     0
##
## affected :
```

```
##           mean    sd Min 2.5% 25% 50%    75% 97.5% Max  nsv Na's
## median 0.1518 0.359   0    0   0   0 0.0000     1   1 1001    0
## mean   0.1548 0.353   0    0   0   0 0.0589     1   1 1001    0
## 2.5%   0.0599 0.237   0    0   0   0 0.0000     1   1 1001    0
## 97.5%  0.2707 0.445   0    0   0   0 1.0000     1   1 1001    0
```
